# Multi-omics and machine learning based on clinical samples: revealing the toxicity characteristics in adverse reactions to *Evodia rutaecarpa*

**DOI:** 10.3389/fphar.2026.1901889

**Published:** 2026-10-19

**Authors:** Xiyue Tan, Jin Xie, Xiaochuan Guo, Lv Wu, Senpan Shi, Lingping Fu, Wenwen Zhang, Lingshun Zeng, Xiao Ma, Jinhao Zeng, Yanling Zhao

**Affiliations:** 1 Chinese Medicine Germplasm Resources Innovation and Effective Uses Key Laboratory of Sichuan Province, School of Pharmacy, Chengdu University of Traditional Chinese Medicine, Chengdu, China; 2 Key Laboratory of Standardization of Chinese Medicine (Chengdu University of Traditional Chinese Medicine), Ministry of Education, Chengdu, China; 3 Department of Pharmacy, Chinese PLA General Hospital, Beijing, China; 4 Lung Disease Diagnosis and Treatment Center, National Medical Center, The First Affiliated Hospital of Henan University of Chinese Medicine, Zhengzhou, China; 5 Collaborative Innovation Center for Chinese Medicine and Respiratory Diseases Co-constructed by Henan Province & Education Ministry of P.R. China / Henan Key Laboratory of Chinese Medicine for Respiratory Diseases, Henan University of Chinese Medicine, Zhengzhou, China; 6 The First Clinical Medical College, Henan University of Chinese Medicine, Zhengzhou, China; 7 College of Medicine and Life Sciences, Chengdu University of Traditional Chinese Medicine, Chengdu, China; 8 Hospital of Chengdu University of Traditional Chinese Medicine, School of Clinical Medicine, Chengdu University of Traditional Chinese Medicine, Chengdu, China

**Keywords:** adverse reactions, *Evodia rutaecarpa*, machine learning, mild toxicity, multi-omics

## Abstract

**Background:**

*Evodia rutaecarpa* (EVR) is a widely used low-toxicity traditional Chinese medicine in clinical practice. Its adverse reactions are often subtle with prominent inter-individual variability, and the associated risks are frequently underestimated. This study conducted multi-omics analysis of the metabolic and microbiological characteristics of its related adverse reactions, and constructed a prediction model to provide a reference for individualized safe medication.

**Methods:**

86 patients with gastric diseases receiving EVR-containing compound formulas were enrolled and divided into a control group (n = 50) and an adverse reaction group (n = 36) based on the presence or absence of adverse reactions. Serum, urine, and saliva samples were collected for untargeted metabolomics and 16S rRNA sequencing, respectively. Seven machine learning algorithms (logistic regression, random forest, LightGBM, *etc.*) were employed to construct predictive models in combination with LOOCV, screen key features, and derive a multi-omics joint prediction formula.

**Results:**

Clinically, adverse events related to EVR predominantly presented as upper-gastrointestinal symptoms. Omics profiling of patient samples identifies tentative correlative trends between EVR-associated adverse events and systemic changes including disturbed amino acid metabolism, shifted neuroactive molecule profiles, altered bile secretion signatures and oral microbiota dysbiosis. Machine learning screening yields multiple model-associated classification features from multi-omics datasets. Serum PE (P-18:1/22:0), hydroxyisocaproic acid and ureidoisobutyric acid show statistical trends linked to lower model prediction outputs for adverse events. Urinary 1, 3-Dihydro-(2H)-indol-2-one and MG (0:0/20:4/0:0) correlate with reduced predictive values, whereas arachidoyl ethanolamide associates with elevated model prediction scores. Higher relative abundances of *Granulicatella*, *Alysiella*, *Leptotrichia hongkongensis* and *Streptococcus* in saliva correspond to higher predictive probabilities from the classification model. An AUC-weighted multi-omics joint formula is established to calculate individual predictive likelihood of EVR-related adverse reactions. Scores ≥ 0.597 indicate higher model-derived predictive tendency, while scores below 0.597 indicate lower predictive possibility.

**Conclusion:**

The ten omics-derived discriminative features and the multi-omics joint prediction formula developed in this work enable quantitative estimation of individual-level risk for EVR-related adverse reactions. They offer preliminary references for safety assessment, risk stratification, and personalized application of low-toxicity traditional Chinese medicines.

## 1 Introduction

Safety research and surveillance of traditional Chinese medicine have long focused on drugs listed in the pharmacopoeias as highly toxic or toxic. This is because the risks of these drugs are clearly defined, making related events easier to identify and track (Sharif et al., 2024). In contrast, mildly toxic or low-toxicity drugs listed in pharmacopoeias are often underestimated in clinical practice due to their wider range of applications, less severe and scattered adverse reactions, and significant individual differences. The risk of these drugs may not necessarily manifest as acute organ damage, but rather as low-level cumulative metabolic stress and related discomfort symptoms. Therefore, it is crucial to develop a research strategy that can not only systematically observe the overall changes in metabolic networks, but also screen for robust feature combinations in highly heterogeneous samples.

Among these mildly toxic traditional Chinese medicines, *Evodia rutaecarpa* (EVR) is a typical representative (Zhang et al., 2021a). EVR is the mature fruit of plants of the genus *Evodia* in the *Rutaceae* family, and is a widely used traditional medicine (Cao et al., 2019; Su et al., 2022). EVR is mainly distributed in East Asia (such as China, Japan, and South Korea) as well as some temperate and tropical regions. EVR and its extracts have various biological activities, including analgesic, anti-inflammatory, gastrointestinal motility-regulating, cardioprotective, antitumor, and antibacterial effects (Liao et al., 2011; Li et al., 2023). In clinical practice, EVR and its formulations are commonly used to treat diseases related to gastrointestinal dysfunction, upper gastrointestinal symptoms, abnormal blood pressure, and certain skin and mucosal inflammations, such as abdominal pain, vomiting, diarrhea, indigestion, hypertension, eczema, and oral ulcers (Chen et al., 2023; Xiao et al., 2023). For example, Wuzhuyu Decoction is often used to alleviate upper gastrointestinal discomfort symptoms such as nausea and vomiting, and its therapeutic effect is believed to comprehensively improve the disease state accompanied by digestive symptoms (Song et al., 2025). Zuojin pill and its modified formula have long been used in clinical prescriptions for gastrointestinal discomfort, and have been evaluated in modern studies for their effects on gastrointestinal dysfunction and chronic gastric mucosal lesion-related phenotypes (Wang et al., 2023; Liu S et al., 2025). In addition, Sishen Pill has been widely used in the study of intestinal inflammation and mucosal barrier damage, and has shown certain therapeutic effects. These clinical applications demonstrate its therapeutic potential in cases of chronic diarrhea and disruption of gut homeostasis (Liu H et al., 2025). EVR has become an important and commonly used option in the treatment of gastrointestinal and other diseases.

However, with the expanding clinical utilization of EVR, safety-related concerns have grown. Although traditional literature classifies it as a mildly toxic substance, existing research suggests that its use may still pose certain safety risks (Zhang et al., 2021b; Yan et al., 2023). Clinical adverse events linked to EVR can include abdominal pain, vomiting and blurred vision (Xiao et al., 2023). A systematic study of organ-specific toxicity suggests that EVR toxicity is not confined to a single organ but mainly affects the liver, spleen, lungs, kidneys, and central nervous system. Meanwhile, multiple molecular monomers in EVR have also been shown to have toxic effects (Li et al., 2025). Researchers have pointed out that the main active ingredient of EVR mediates liver and cardiac toxicity by inducing oxidative stress and mitochondrial dysfunction at the monomer level (Yang et al., 2017; Li et al., 2019). In addition, certain bioactive compounds containing indole or furan rings in EVR may form reactive metabolic intermediates during *in vivo* metabolism, thereby inducing liver toxicity (Zhang et al., 2020). Although studies have reported the toxicity of EVR or related natural products, they are mostly based on a single molecular perspective or partial organ characterization, making it difficult to systematically reflect early molecular events and individual differences in toxicity occurrence.

This study selected patients receiving EVR-containing compound formulas therapy as the study subjects and compared the differences in metabolic and microbiome profiles between the control group and the adverse reaction group. Further integrate machine learning technologies to identify key metabolites and microorganisms closely associated with adverse reaction risks, and establish personalized risk prediction models. The experimental procedure is illustrated in Figure 1. Through this study, we aimed to identify biological features associated with EVR-related adverse reactions and to provide new research strategies for the safety evaluation of low-toxicity traditional Chinese medicines and personalized clinical treatment.

**FIGURE 1:**
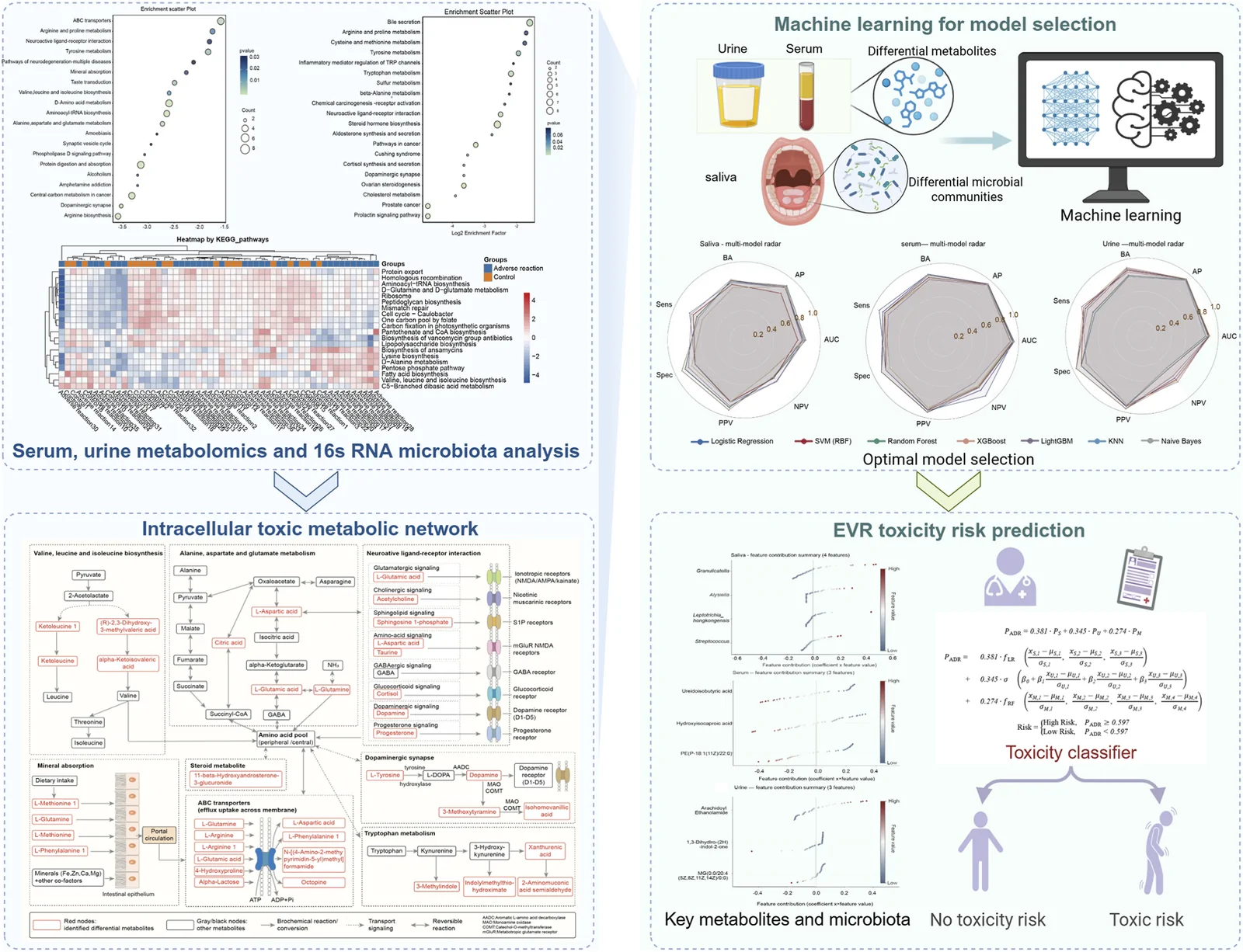
Experimental flowchart Samples from patients are collected for serum metabolomics, urine metabolomics, and 16S rRNA microbiome analysis to obtain the metabolic network in the body. Then, model-associated discriminative molecular features are screened through machine learning, and a risk prediction model is constructed for clinical toxicity prediction. Created in BioRender. Xiyue, T. (2026) https://BioRender.com/ahclk8v.

## 2 Methods

### 2.1 Composition analysis of the water extract of EVR

The EVR material was first extracted with water. The aqueous extract was then subjected to compositional analysis by UHPLC-HRMS, following pre-treatment as described in the Supplementary Methods. The main chemical components were then detected using chromatographic analysis methods. Finally, the representative components were preliminarily identified by integrating retention times, mass spectrometry data, database matching, and relevant literature. Detailed protocols for sample preparation, chromatographic conditions, mass spectrometry parameters, and component identification methods are provided in the Supplementary Methods section.

### 2.2 Patient recruitment and experimental design

This study was reviewed and approved by the Medical Ethics Committee of the Affiliated Hospital of Chengdu University of Traditional Chinese Medicine (Approval No. 2023KL-028). Saliva, blood, and urine samples were collected. 86 patients with gastric diseases were included in this study, all of whom received EVR-containing compound formulas. The patients were grouped according to whether there were adverse reactions after EVR-containing compound formulas administration: the control group included 50 patients who did not experience any adverse reactions, while the adverse reaction group included 36 patients who experienced adverse reactions. Not all of the 86 patients provided all three types of samples simultaneously. All collected samples were stored at -80 °C for future untargeted metabolomics and 16S rRNA microbiota analysis.

#### 2.2.1 Inclusion criteria

1) Age ≥ 18 years old; 2) Attended the outpatient or inpatient departments of the hospital and were able to complete medical interviews and sample collection; 3) Adverse reaction group: with a clear history of using EVR-containing compound formulas, and subsequent symptoms that meet the research definition of adverse reactions; 4) Control group: There is a clear history of using EVR-containing compound formulas therapy, and no symptoms that meet the definition of adverse reactions in the study have occurred subsequently. 5) Sign the informed consent form and be willing to cooperate with the research process.

#### 2.2.2 Exclusion criteria

1) Concurrent severe liver and kidney dysfunction or other major organ system failure; 2) Use antibiotics, probiotics/synbiotics, oral antibacterial mouthwash, or treatments that affect the microbiota in the past 4 weeks; 3) Acute infection, major surgery, severe trauma, or acute stress events within the past 3 months; 4) Concurrent malignant tumors, active autoimmune diseases, or long-term immunosuppressive therapy; 5) Pregnancy or lactation period; 6) Other factors that the researchers believe may affect the interpretation of the outcome, such as non-compliant sample collection, missing data, *etc.*

#### 2.2.3 Adverse reaction assessment and patient follow-up

Adverse reactions are defined as upper gastrointestinal discomfort symptoms in subjects after taking EVR-containing compound formulas, mainly including heartburn, chest tightness, and the occurrence or aggravation of gastric reflux. All symptoms and cases of adverse reactions were independently evaluated by two or more associate chief physicians. Cases in which the two physicians disagreed were resolved through joint discussion before a consensus was reached.

#### 2.2.4 Sample collection and storage

All subjects were asked to provide fasting venous blood, morning urine, and unstimulated saliva. Venous blood and morning urine were collected in the morning after an overnight fast; serum was separated and stored at -80 °C. Saliva was collected after fasting for 1 h, into sterile centrifuge tubes, and stored at -80 °C until analysis.

### 2.3 Untargeted metabolomics analysis of plasma and urine

#### 2.3.1 Sample pretreatment

A 100 μL aliquot of each plasma or urine sample was mixed with 400 μL of a pre-cooled mixture (methanol/acetonitrile = 3:1) at -40 °C for protein precipitation. After vortexing for 5 min and ultrasonication for 15 min, the mixture was allowed to stand in an ice bath at 4 °C for 1 h. The samples were then centrifuged at 12,000 rpm and 4 °C for 15 min. Subsequently, 100 μL of the supernatant from each sample was collected and concentrated to dryness under vacuum. The dried residue was reconstituted in 50 μL of 50% methanol water (1:1), vortexed, and centrifuged. After filtration through a 0.22 μm membrane, the filtrate was collected for subsequent analysis.

#### 2.3.2 Liquid chromatography-mass spectrometry analysis

Liquid chromatography mass spectrometry analysis was performed using a Thermo Vanquish liquid chromatography system coupled to a Thermo Q Exactive HF-X high-resolution mass spectrometer. Chromatographic separation was performed on an HSS T3 column measuring 2.1 mm × 100 mm with a particle size of 1.8 μm. The mobile phases consisted of water containing 0.1% formic acid as mobile phase A and acetonitrile containing 0.1% formic acid as mobile phase B. The proportion of mobile phase B was maintained at 2% for the first 0.5 min, increased to 50% at 2 min and to 98% at 5 min, maintained at 98% until 8 min, decreased to 2% at 10 min, and maintained at 2% until 12 min. The flow rate was 0.3 mL/min, the column temperature was maintained at 40 °C, and the injection volume was 2 μL. Mass spectrometric data were acquired in both positive and negative ion modes, with spray voltages of 3.6 kV and −3.6 kV, respectively. The capillary temperature was maintained at 325 °C, and the mass scan range was m/z 70–1,050. Data were acquired in full-scan mode combined with data-dependent acquisition. Quality control samples were prepared by mixing equal volumes of all study samples, and one quality control sample was injected after every 10 study samples. Before formal sample analysis, five consecutive quality control samples were injected to assess system stability, and formal analysis was initiated when the relative standard deviation of the signal intensity was no greater than 10%.

#### 2.3.3 Data processing and multivariate statistical analysis

The raw data was subjected to peak recognition, alignment, extraction, and normalization using Compound Discoverer 3.3. Alignment Model = Adaptive curve, Maximum Shift = 0.5 min,Mass Tolerance = 10 ppm,Intensity Tolerance = 30%,S/N Threshold = 1.5. Metabolites with < 20% missing values were imputed with linear regression, while the rest were excluded. Samples were normalized to the median of the maximum peak areas across all samples. Compound identification was based on exact mass, retention time, and MS/MS spectra, cross-checked against mzCloud, ChemSpider, KEGG, HMDB, and mzVault, and graded according to MSI standards. Results from the positive and negative ion modes were then combined, retaining the larger total peak area for identical metabolites. Only metabolites with RSD ≤ 30% and detected in ≥ 80% of QC samples are retained for statistical purposes. PCA and OPLS-DA/PLS-DA were used for unsupervised and supervised discrimination and validation, respectively, and model validity was assessed using R2X, R2Y, Q2Y, the score plot, and 200 permutation tests (Q2 intercept < 0.05). Differential metabolite screening: Two groups were compared based on OPLS-DA VIP, combined with independent sample t-test P-value (Benjamini-Hochberg correction, FDR < 0.05) and fold change.

### 2.4 Saliva 16S rRNA gene sequencing and bioinformatics analysis

#### 2.4.1 Genomic DNA extraction and amplification

Extract total DNA of saliva microorganisms using QIAamp DNA Stool Mini Kit. After quality control by agarose gel electrophoresis and NanoDrop (concentration ≥ 20 ng/μL; OD260/280 = 1.8–2.0), the DNA was stored for subsequent amplification. Amplify the V3-V4 region using primers 338F (5′-ACT​CCT​ACG​GGA​GGC​AGC​A-3′) and 806R (5′-GGACTACHVGGGTWTCTAAT-3′). After library construction, Illumina NovaSeq 6,000 and PE250 strategy sequencing were performed.

#### 2.4.2 Sequencing and data processing

Raw data quality control: Remove reads with low-quality bases (Q ≤ 15) exceeding 40 bp, reads containing N bases > 10 bp, and sequences overlapping with adapters > 10 bp. Check and ensure that the error rate of each point is < 1%, and the Q30 proportion is ≥ 80%. Subsequently, QIIME 2 was used for downstream processing, and DADA2 was used for filtering, chimera removal, paired-end merging, and denoising, from which amplicon sequence variants (ASVs) were generated. Annotate the q2 feature classifier trained with Silva-138–99 Naive Bayes classifier and obtain the ASV table.

#### 2.4.3 Diversity and functional prediction analysis

Alpha diversity (observed, Chao1, Shannon) was calculated, and rarefaction curves were plotted. The beta diversity was analyzed using Bray-Curtis and weighted/unweighted UniFrac, PCA/NMDS/PCoA dimensionality reduction, ANOSIM and Adonis tests for inter group differences, and a phylogenetic tree was constructed. Differential abundance analysis was performed using LEfSe (Kruskal Wallis and Wilcoxon, LDA). Functional prediction was performed using PICRUSt2 to infer the potential abundance of MetaCyc, EC, COG, and KEGG pathways based on ASV inference. Statistical significance threshold p < 0.05, Benjamini Hochberg corrected FDR < 0.05.

### 2.5 Integrated analysis of statistics and multi-omics

All statistical analyses were performed using R software. Normal distribution data is analyzed using t-test or ANOVA, while non-normal data is analyzed using Mann Whitney U or Kruskal-Wallis. Multiple comparisons were performed using Benjamini-Hochberg correction (FDR < 0.05). The correlation between metabolites was analyzed using Pearson correlation analysis and visualized using a network. Enrichment at the pathway level was evaluated using KEGG and the significance of enrichment was assessed using hypergeometric tests.

### 2.6 Machine learning

To evaluate the predictive performance of different machine learning algorithms on small sample multi-omics data, we performed a side-by-side comparison of seven commonly used classifiers under a unified feature-selection pipeline (Welch’s t-test prescreening → L1 regularized embedded selection). Each model shares the same selected features (3 in serum and urine, 4 in saliva) at each fold in Leave One Out Cross Validation (LOOCV), only replacing the final classifier to ensure fairness in comparison. Considering the limited sample size, this study adopts the LOOCV method to evaluate the performance of the model. All feature selection steps are performed within the crossvalidation loop to avoid information leakage. Due to the imbalanced sample size between the adverse reaction group and the control group, this study adopted a category weight adjustment strategy (class_weight = 'balanced’) in model training to give higher weights to minority classes and balance the classifier’s attention to both classes. The Youden’s J statistic (J = Sensitivity + Specificity-1) was used to determine the optimal classification threshold that best balances sensitivity and specificity. The performance evaluation indicators of the model include: AUC-ROC (area under the receiver operating characteristic curve), sensitivity, specificity, positive predictive value (PPV), negative predictive value (NPV), balance accuracy, and Brier score. The omics weights were calculated based on the AUC values of the omics deployment models. This study uses visualization methods similar to SHAP (SHapley Additive exPlanations) (beeswarm plot and waterfall plot) to display feature contributions, in order to visually present the risk factor composition of individual patients.

Detailed procedures for exogenous metabolite removal, feature pre-screening, model hyperparameters, regularized logistic regression, LOOCV implementation, feature importance analysis, and individualized prediction interpretation are provided in the Supplementary Methods.

## 3 Results

### 3.1 Component analysis of the aqueous extract of EVR

The aqueous extract of EVR was analyzed by UHPLC-HRMS in full-scan mode in both positive and negative ion modes, yielding the base-peak ion chromatograms shown in Supplementary Figure S1. Molecular ion peaks, secondary fragmentation information and retention times were then compared against standard spectral libraries. In combination with literature reports, the major components in the EVR water extract were identified, yielding 13 representative compounds (Supplementary Figure S1). The main components were flavonoids (e.g., quercetin, isorhamnetin, catechin and hesperidin), phenolic acids (e.g., neochlorogenic acid, chlorogenic acid, caffeic acid and gallic acid), organic acids (e.g., citric acid), coumarins, aldehydes (e.g., trans-cinnamaldehyde) and alkaloids (e.g., rutaecarpine and dehydroevodiamine). Relevant information was summarized in Supplementary Table S1.

### 3.2 Patient baseline information

Between 2024 and 2025, this study screened patients through communication with them at the outpatient and inpatient departments of the Digestive Medicine Department of the Affiliated Hospital of Chengdu University of Traditional Chinese Medicine, and ultimately collected samples after obtaining informed consent. The experimental procedure is shown in Figure 2. The study included a total of 86 patients aged 23–75 years with a diagnosis of chronic gastritis-related diseases. Based on the comparison of medical records, it was confirmed that the patient had taken EVR-containing compound formulas recently. Based on whether patients experienced adverse reactions after taking EVR-containing compound formulas, they were divided into two groups: the control group and the adverse reaction group. Adverse reactions mainly manifested as gastrointestinal discomfort, such as heartburn, dry mouth, a bitter taste, chest tightness and acid reflux. The average ages of the adverse reaction group and the control group were 53.4 ± 11.8 years and 50.2 ± 12.0 years, respectively. There were no statistically significant differences in gender distribution and diagnosed diseases (Table 1). The baseline characteristics of the two groups are comparable. Of the enrolled patients, 56 provided saliva samples for 16S rRNA sequencing of the oral microbiota (21 control, 35 adverse reaction), 48 provided urine samples for urinary metabolomics (13 control, 35 adverse reaction), and serum samples from a further 52 patients were used for serum metabolomics (16 control, 36 adverse reaction).

**FIGURE 2:**
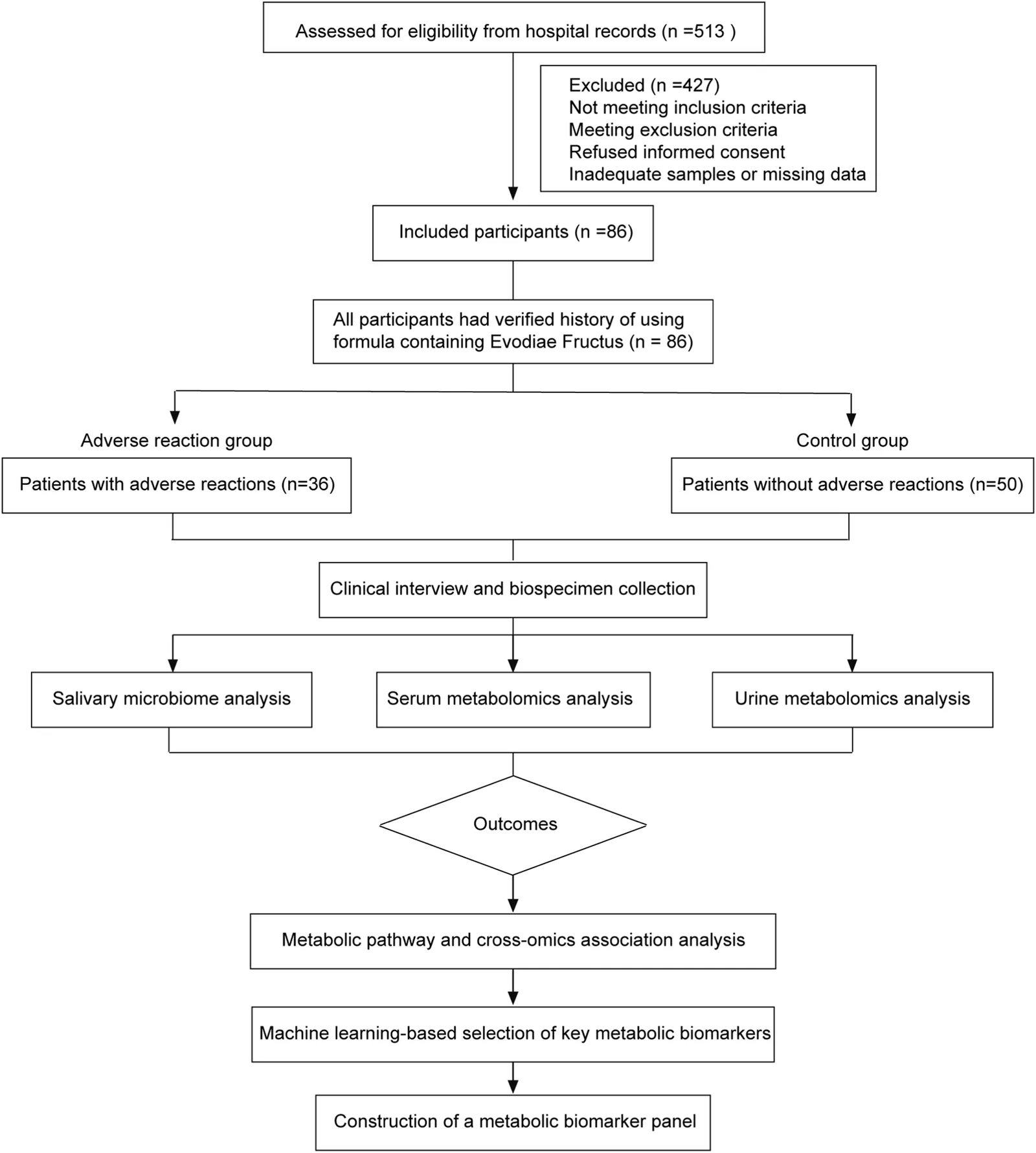
Study design and participant flow diagram After screening, 86 participants with verified use of EVR–containing formula were enrolled and divided into adverse reaction group (n = 36) and control group (n = 50), followed by multi-omics analysis and machine learning to construct a metabolic biomarker panel.

**TABLE 1:** Patient baseline data.

Variable	Total sample (N = 86)	Adverse reaction group (N = 36)	Control group (N = 50)
Age (years), mean ± SD	51.6 ± 12.1	53.4 ± 11.8	50.2 ± 12.0
Sex, n (%)	​	​	​
Male	42 (48.8%)	13 (36.1%)	29 (58.0%)
Female	44 (51.2%)	23 (63.9%)	21 (42.0%)
Primary diagnosed disease, n (%)	​	​	​
Chronic atrophic gastritis	71 (82.6%)	30 (83.3%)	41 (82.0%)
Metaplasia of the gastric mucosal epithelium	40 (46.5%)	18 (50.0%)	22 (44.0%)
Gastro-oesophageal reflux	32 (37.2%)	15 (41.7%)	17 (34.0%)

### 3.3 Serum metabolomics analysis of EVR adverse reactions

To systematically evaluate the serum metabolic differences between the adverse reaction group and the control group after taking EVR-containing compound formulas, we conducted a untargeted metabolomic analysis on the serum of 52 patients. The samples were analyzed by LC-MS in both positive and negative ion modes. The TIC was shown in Figures 3A,B. Permutation tests were performed using OPLS-DA to verify the validity of the model (Figures 3D,G). The results show that all *R* ^2^ and Q^2^ values on the left are lower than those of the original data points on the right, indicating that the OPLS-DA model is not overfitted and is valid. An S-plot (Figures 3E,H) was used to identify the metabolic features contributing most to the inter-group differences. Furthermore, as shown in the volcano plot, in negative ion mode, 411 metabolites were downregulated and 418 were upregulated in the adverse reaction group compared with the control group (Figure 3I). In contrast, in positive ion mode (Figure 3J), 261 metabolites were downregulated and 673 were upregulated. We matched the metabolites identified in the positive and negative ion modes against the KEGG database and annotated the KEGG pathways in which they are involved. We selected the top 20 pathways with the highest p-values for presentation (Figure 3K). The results revealed significant enrichment in pathways such as ABC transporters, arginine and proline metabolism, and neuroactive ligand-receptor interaction. Other significantly enriched pathways include Tyrosine metabolism, Valine, leucine and isoleucine biosynthesis, Aminoacyl-tRNA biosynthesis, *etc.* Overall, multiple pathways related to amino acid metabolism and neuroactivity exhibited correlative alterations in patients with adverse reactions.

**FIGURE 3:**
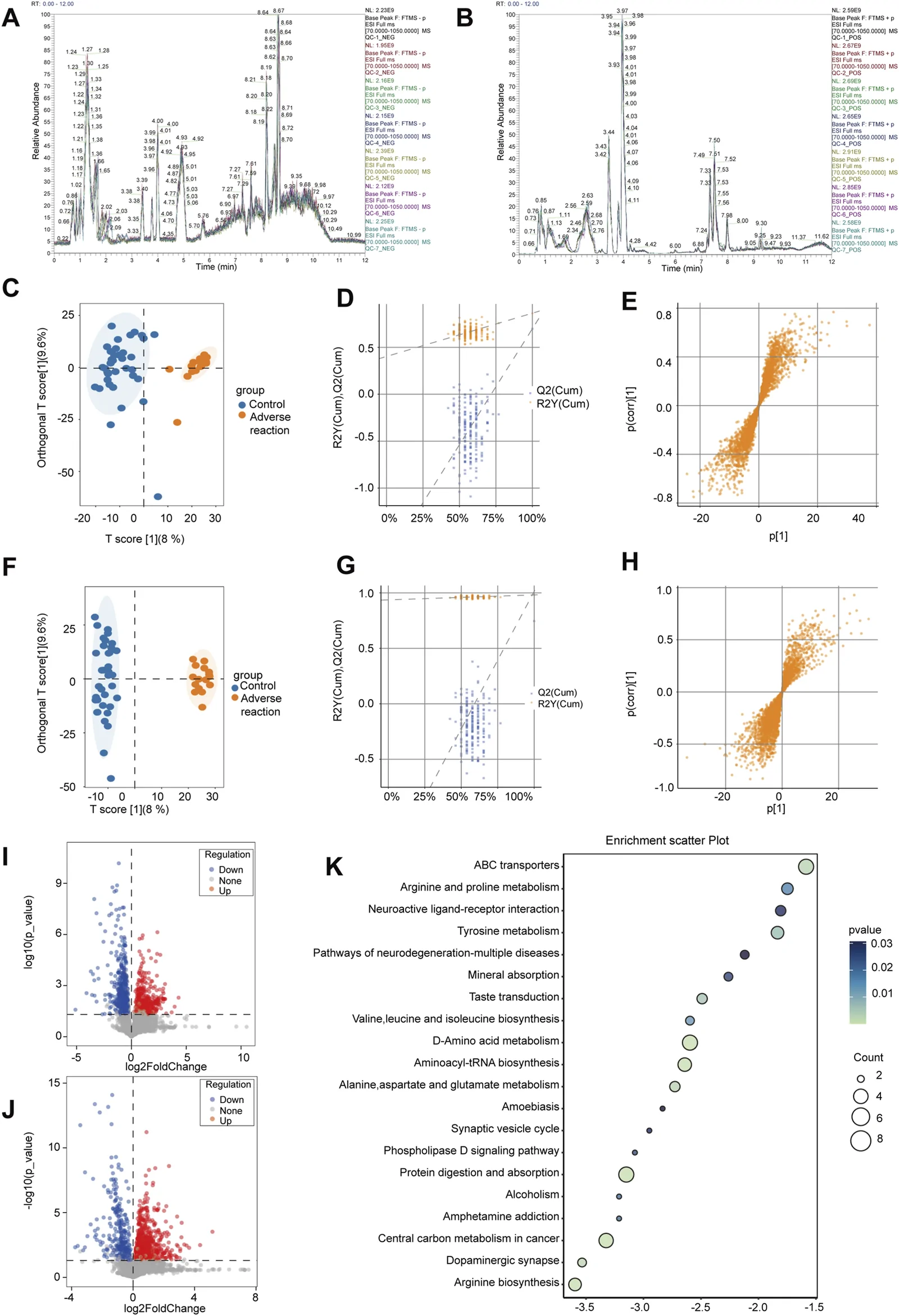
Serum metabolomics analysis of EVR adverse reactions **(A,B)** positive and negative ion mode detection, Total Ion Chromatography (TIC); **(C)** scatter plot obtained by OPLS-DA in negative mode; **(D)** OPLS-DA permutation test in negative mode; **(E)** S-plot in negative mode; **(F)** scatter plot obtained by OPLS-DA in positive mode; **(G)** OPLS-DA replacement test in positive mode; **(H)** S-plot in positive mode; **(I)** volcano plot of differentially expressed metabolites in negative mode; **(J)** volcano plot of differentially expressed metabolites in positive mode; **(K)** KEGG pathway enrichment analysis of differentially expressed metabolites.

### 3.4 Urine metabolomics analysis of EVR adverse reactions

We next performed untargeted metabolomic profiling of urine samples in both positive and negative ion modes to characterize the urinary metabolic alterations associated with EVR-containing compound formulas. The TICs in positive and negative ion modes are shown in Figures 4A,B, respectively. The scatterplot of OPLS-DA scores (Figures 4C,F) revealed a certain separation trend between the two groups of samples in both negative and positive ion modes. Through OPLS-DA permutation test analysis, the models under both modes were not overfitted and exhibited good predictive ability. In negative ion mode, compared with the control group, there were 157 metabolites upregulated and 417 metabolites downregulated in the adverse reaction group (Figure 4I). In the positive ion mode, 513 metabolites were downregulated and 246 metabolites were upregulated (Figure 4J). KEGG enrichment analysis (Figure 4K) showed that the differential metabolites were mainly enriched in pathways such as bile secretion, arginine and proline metabolism, cysteine and methionine metabolism, and tyrosine metabolism. The results showed that the differential metabolites were primarily enriched in pathways such as bile secretion, arginine and proline metabolism, cysteine and methionine metabolism, and tyrosine metabolism. Overall, correlative shifts in urinary metabolism among patients with adverse reactions were mainly enriched in pathways related to bile acid metabolism, various amino acid metabolisms, and neural signal transduction.

**FIGURE 4:**
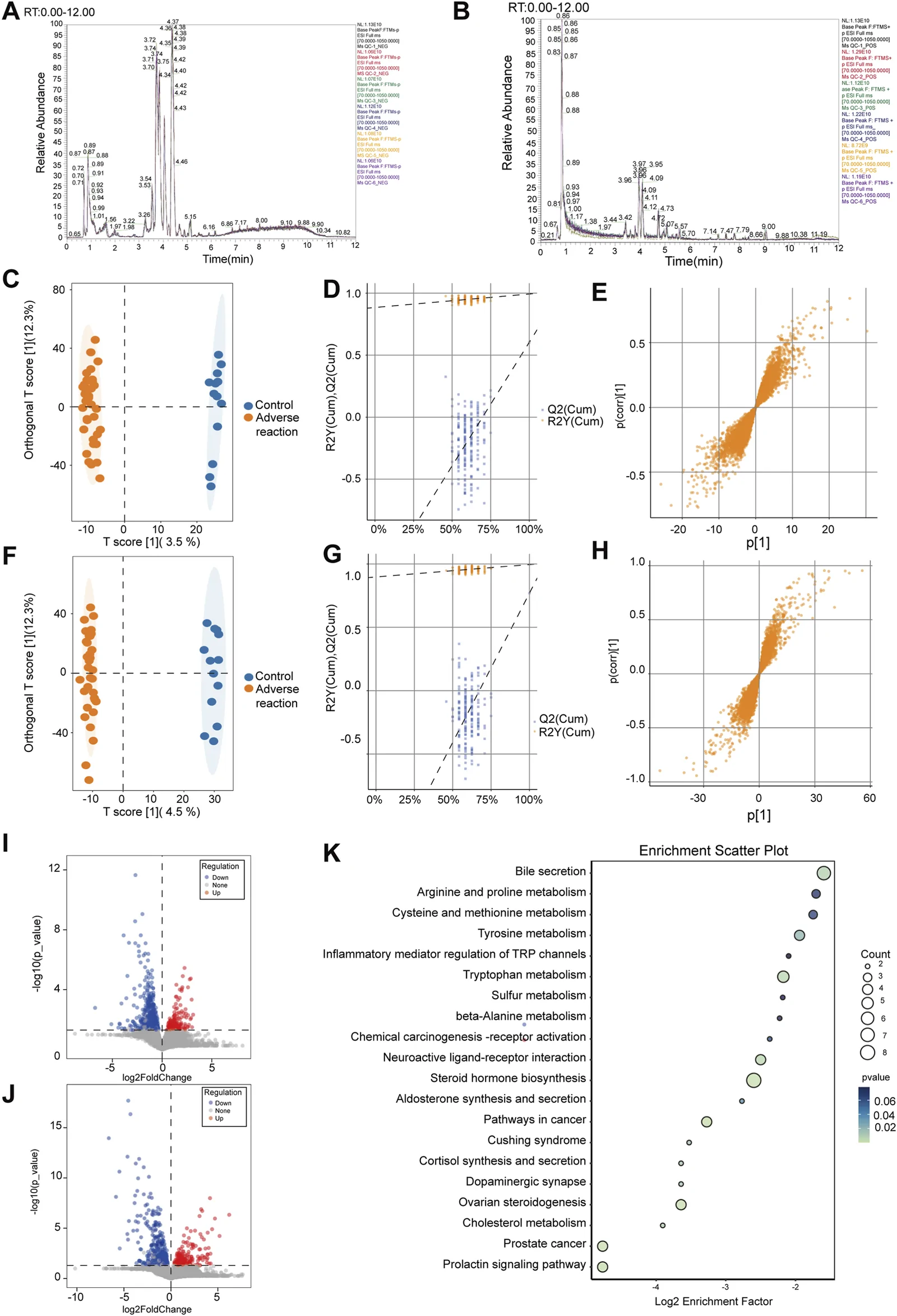
Urinary metabolomics analysis **(A,B)** TIC spectra in positive and negative ion modes; **(C)** OPLS-DA scatter plot in negative ion mode; **(D)** OPLS-DA replacement test in negative ion mode; **(E)** S-plot in negative ion; **(F)** OPLS-DA scatter plot in positive ion mode; **(G)** OPLS-DA replacement test in positive ion; **(H)** S-plot in positive ion; **(I)** volcano plot of differentially expressed metabolites in negative ion; **(J)** volcano plot of differentially expressed metabolites in positive ion; **(K)** KEGG pathway enrichment analysis of differentially expressed metabolites.

### 3.5 Salivary microbiome analysis of EVR adverse reactions

Saliva samples from 56 patients were collected for 16S rRNA sequencing in this experiment. In terms of α diversity, there were no significant differences in the Chao1 or ACE index compared with the control group (Figures 5A,C). The Simpson index was significantly reduced in the adverse reaction group (*P* = 0.013) (Figure 5D). The evenness of the microbiota in the adverse reaction group decreased, with a more concentrated dominant microbiota. The Shannon index showed a slight decrease (Figure 5B). In the β-diversity analysis, Principal Coordinate Analysis (PCoA) of the Bray-Curtis distance revealed (Figure 5F) that the adverse reaction group and the control group overlapped considerably in the principal coordinate space, with only a slight shift in the centroid position. ANOSIM analysis revealed few differences in the overall structure of the salivary microbiota between the two groups (Figure 5E). The Venn diagram results indicated that the differences in microbial diversity between groups may be even more significant. We identified species with significant differences in abundance between the two groups through LEfSe analysis (LDA > 2) (Figure 5H). The results indicated that, compared with the control group, the adverse reaction group showed increased relative abundances of *Actinobacteriota*, *Corynebacterium*, *Rothia* and *Streptococcus*. Furthermore, we utilized PICRUSt2 to predict the function of the microbiota. The KEGG pathway enrichment results (Figure 5I) suggested enhanced enrichment of pathways related to amino acid metabolism (such as arginine, proline, and tryptophan metabolism), bacterial chemotaxis, and biofilm formation in the adverse reaction group, while reduced enrichment was observed for pathways involved in short-chain fatty acid biosynthesis. Overall, distinct oral microbial compositional shifts were observed in patients with adverse reactions. Shifts in microbial composition and functional profiles at the genus level might go along with the presence of adverse gastrointestinal manifestations.

**FIGURE 5:**
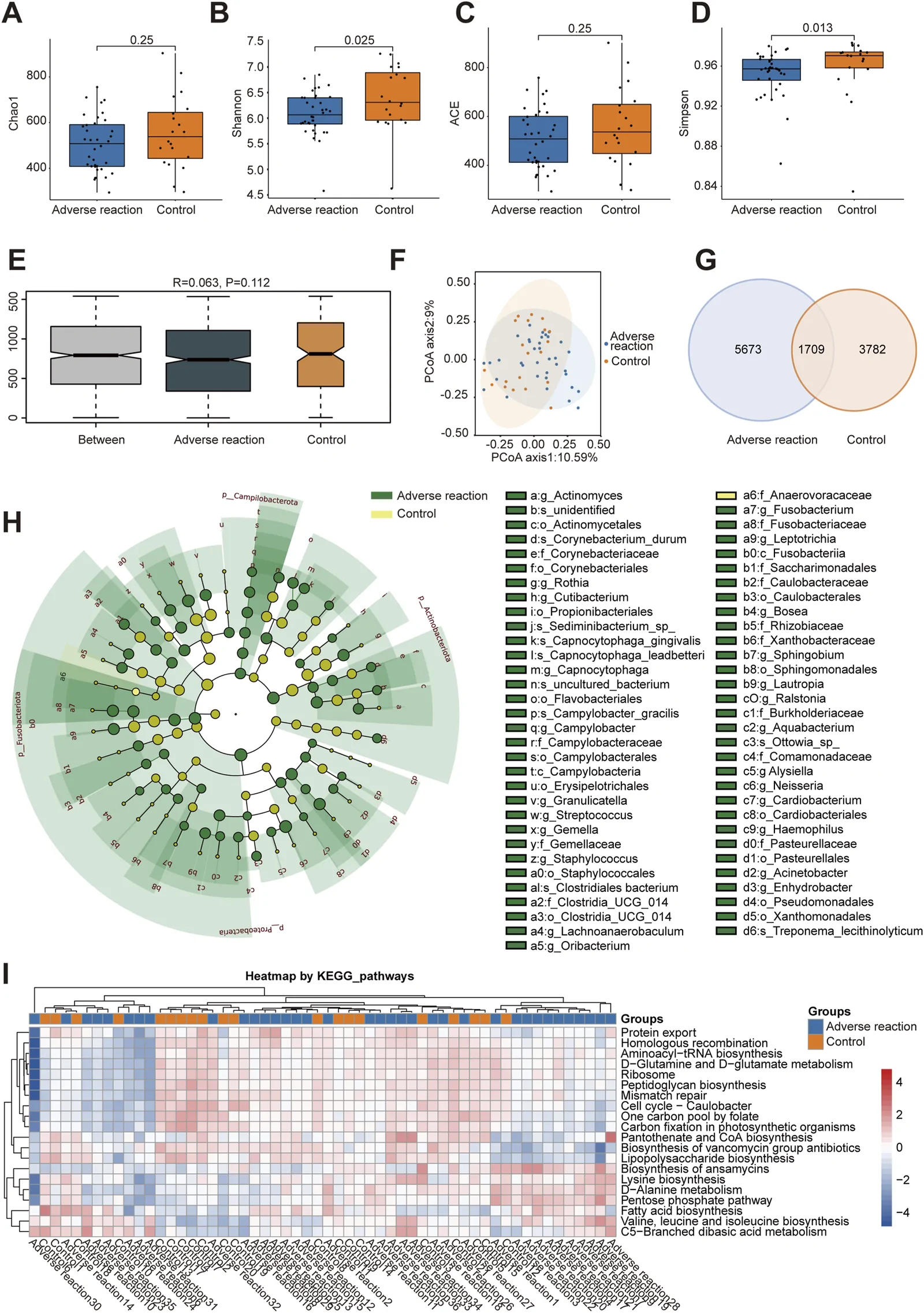
Comparison of Salivary Microbiota Diversity and Community Structure **(A)** comparison of chao1 diversity index; **(B)** comparison of shannon index; **(C)** comparison of ACE diversity index; **(D)** comparison of simpson diversity index; **(E)** ANOSIM analysis results; **(F)** PCoA analysis; **(G)** OTU venn diagram; **(H)** lefse analysis; **(I)** KEGG functional pathway LDA analysis based on PICRUSt.

### 3.6 Overall metabolic network in patients with EVR adverse reactions

After conducting enrichment analysis on urine, serum, and saliva, it was found that after taking EVR, the adverse reactions in the body were mainly concentrated in metabolic aspects related to amino acid metabolism, neural activity, transport, and absorption (Figure 6). Perturbed amino acid pathways included alanine-aspartate-glutamate metabolism, valine/leucine/isoleucine biosynthesis, and tryptophan metabolism. Most amino acids and their intermediate metabolites exhibited elevated abundance in the adverse reaction group, whereas several catabolic products of branched-chain amino acids and downstream metabolites of tryptophan were present at lower levels. Within neuroactivity-related pathways, patients experiencing adverse reactions showed higher levels of metabolites linked to excitatory neurotransmitters and lower levels of molecules associated with inhibitory neurotransmitters and regulatory hormones. The enrichment pattern of ABC transporters and mineral absorption pathways indicated correlated shifts in the transmembrane transport of amino acids, carbohydrates and minerals among affected participants. Meanwhile, there may be coherent and correlated fluctuations in the metabolism of arginine-proline, turnover of branched-chain amino acids, and tryptophan metabolism cascade. The widespread accumulation of amino acid intermediates alongside declines in specific catabolites may point to concurrent changes in substrate utilization, mitochondrial amino acid metabolism, and systemic nitrogen homeostasis. The synchronous changes in metabolites such as glutamate, acetylcholine, dopamine, and cortisol may reflect shifts in gut-brain signaling pathways that could be linked to gastrointestinal discomfort. Altered enrichment of ABC transporter pathways may correspond to distinct transmembrane transport patterns in this cohort, providing a basis for follow-up functional studies.

**FIGURE 6:**
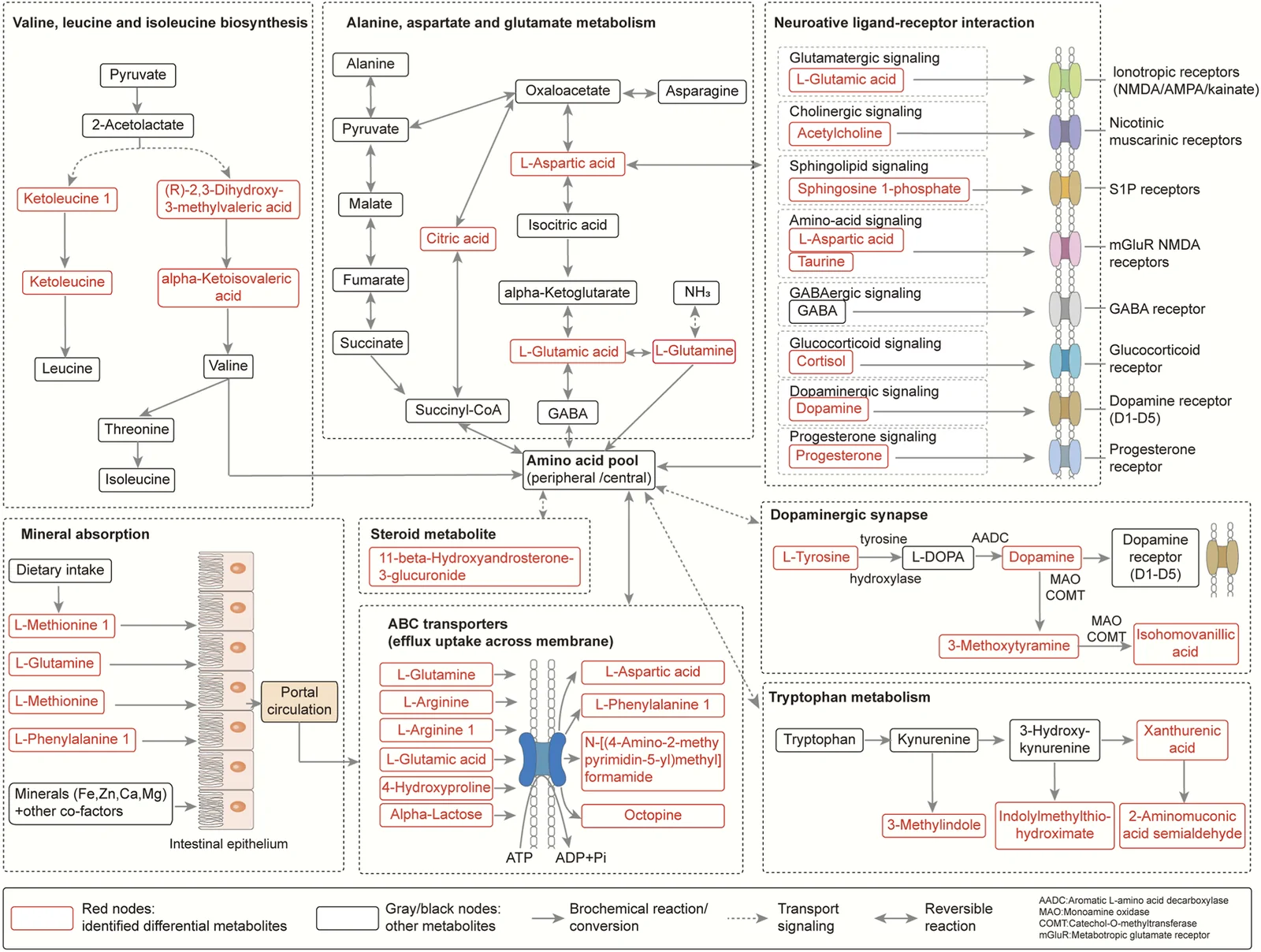
Metabolic network diagram in patients with EVR adverse reactions The major metabolic pathways involved in adverse reaction-related differential metabolites and their interactions, including amino acid metabolism, tryptophan metabolism, branched-chain amino acid biosynthesis, substance transport, and neurotransmitter-related signaling pathways.

### 3.7 Screening and evaluation of predictive performance of machine learning models based on multi-omics data

To systematically evaluate the predictive performance of different machine learning algorithms on small-sample multi-omics data, this study conducted a horizontal comparison of LOOCV for seven commonly used classification models based on a unified feature selection process. Each model shared the same selected features (3 from serum and urine, and 4 from saliva) across all folds, with only the final classifier being replaced to ensure fairness in comparison. For the salivary microbiome (Figure 7A), the random forest model performed best (AUC = 0.842), followed by KNN (0.829), while the baseline LR model achieved an AUC of 0.793 and SVM performed worst. In serum metabolomics (Figure 7B), logistic regression (LR) emerged as the optimal model (AUC = 0.976), followed closely by Naive Bayes (AUC = 0.972), then Random Forest (0.962) and XGBoost (0.955). It is worth noting that the AUC of LR is 0.976, with a specificity of 0.938 and a sensitivity of 0.917, indicating that the true biological signal has been amplified and made linearly distinguishable following denoising. For urine metabolomics (Figure 7C), LightGBM achieved the highest AUC of 0.930. After excluding exogenous metabolites, LightGBM had a sensitivity of 0.886, a specificity of 1.000, and a balanced accuracy of 0.943, SVM (0.925), Naive Bayes (0.921) and LR (0.921) followed closely behind.

**FIGURE 7:**
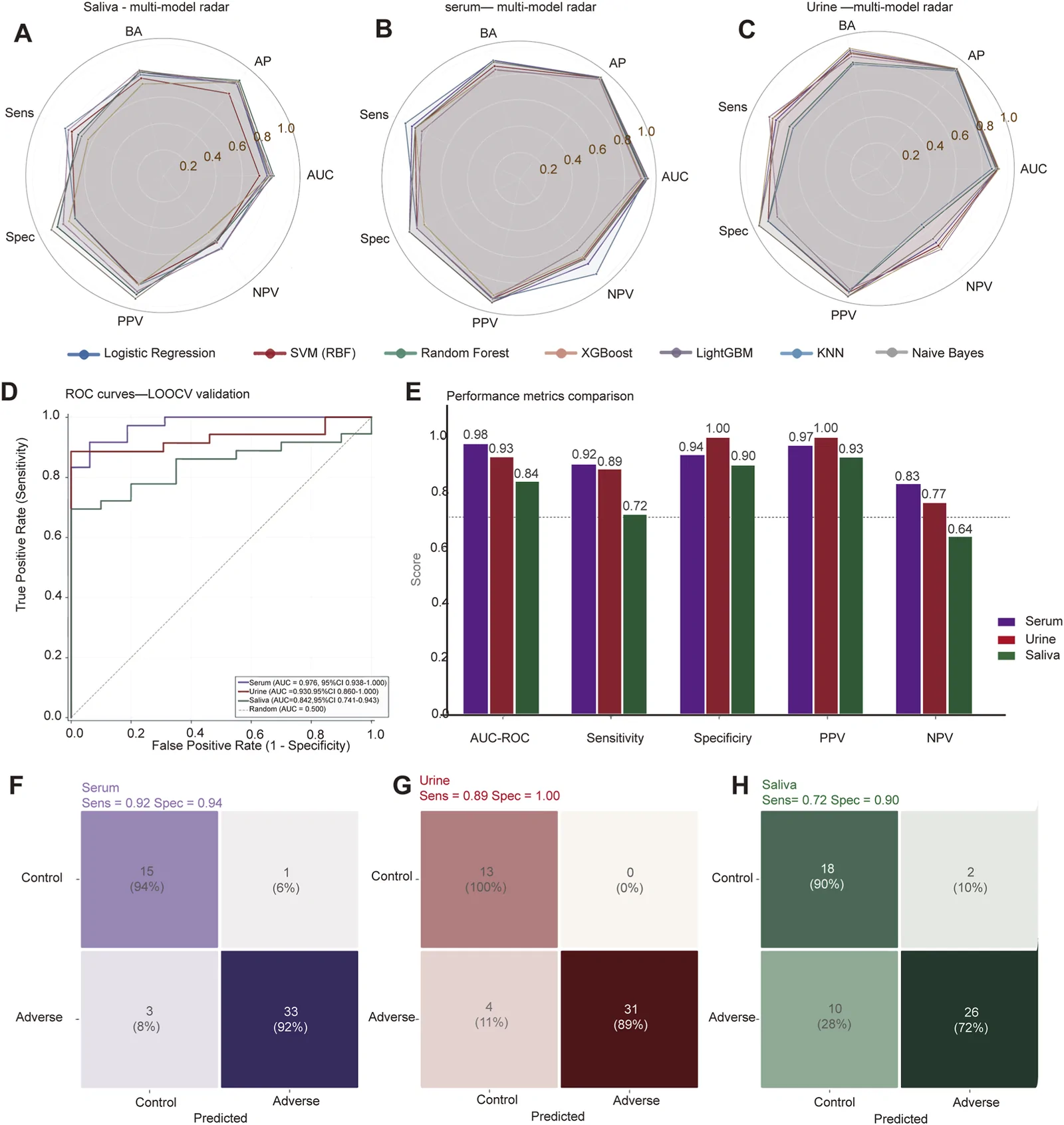
Screening of three omics-based machine learning models **(A)** multi-dimensional performance radar chart for the seven salivary microbiome models; **(B)** multi-dimensional performance radar chart for the seven serum metabolomics models; **(C)** multi-dimensional performance radar chart for the seven urine metabolomics models; **(D)** comparison of ROC curves for the three omics deployment models; **(E)** comparison of multi-indicator performance for the three omics deployment models; **(F)** confusion matrix for the serum metabolomics deployment model; **(G)** confusion matrix for the urine metabolomics deployment model; **(H)** confusion matrix for the saliva microbiome deployment model.

Based on a comparative analysis of the preceding results, the final deployment models for each omics approach were selected to evaluate their performance. The deployment models for the three omics approaches were as follows (Figure 7D): serum metabolomics-LR (AUC = 0.976, 95%CI 0.933–1.000), urinary metabolomics-LightGBM (AUC = 0.930, 95% CI 0.843–0.992), and salivary microbiomics-Random Forest (AUC = 0.842, 95% CI 0.724–0.935). Table 2 summarizes the LOOCV performance metrics of each deployed model. Further comparison of key performance indicators (Figure 7E) showed that the deployed serum metabolomics logistic regression model achieved the highest AUC (0.976), with a sensitivity of 0.917, a specificity of 0.938, and a balanced accuracy of 0.927, indicating a balanced performance across all metrics. The urine metabolomics LightGBM deployment model had an AUC of 0.930, specificity of 1.000, sensitivity of 0.886, balanced accuracy of 0.943, and a Brier score of 0.098. The saliva microbiomics Random Forest deployment model achieved an AUC of 0.842 and had the lowest Brier score (0.163), indicating good calibration of probability predictions. The confusion matrices further confirmed the classification performance of each model: the serum model correctly identified 15 control and 33 adverse-reaction samples, the urine model all 13 control and 31 adverse-reaction samples, and the saliva model 18 control and 26 adverse-reaction samples (Figures 7F–H).

**TABLE 2:** LOOCV performance metrics for omics deployment models across different groups.

Metric	Serum metabolomics (logistic regression)	Urine metabolomics (LightGBM)	Salivary microbiomics (random forest)
AUC-ROC	0.976 (0.933–1.000)	0.930 (0.843–0.992)	0.842 (0.724–0.935)
AUC-PR	0.990 (0.970–1.000)	0.979 (0.947–0.997)	0.930 (0.865–0.974)
Balanced accuracy	0.927 (0.840–0.987)	0.943 (0.886–0.986)	0.811 (0.702–0.905)
Sensitivity	0.917 (0.818–1.000)	0.886 (0.771–0.973)	0.722 (0.568–0.861)
Specificity	0.938 (0.800–1.000)	1.000 (1.000–1.000)	0.900 (0.750–1.000)
Positive predictive value	0.971 (0.903–1.000)	1.000 (1.000–1.000)	0.929 (0.818–1.000)
Negative predictive value	0.833 (0.636–1.000)	0.765 (0.529–0.944)	0.643 (0.452–0.812)
Brier score	0.226 (0.221–0.231)	0.098 (0.048–0.160)	0.163 (0.109–0.226)
Optimal threshold	0.495	0.627	0.700

### 3.8 Screening of key metabolites associated with EVR adverse reactions and construction of predictive models

This study further analyzed the key predictive features and their contributions to model outputs. Based on the analysis of omics weighting derived from the AUC of each omics deployment model (Figures 8A,B), serum metabolomics exhibited the highest relative contribution (38.1%), followed by urine metabolomics (34.5%) and saliva microbiomics (27.4%). The highest contribution of serum metabolomics may reflect the role of serum as a systemic circulating fluid that comprehensively mirrors whole-body metabolic status, while the comparable contribution of urine metabolomics may be explained by the direct representation of drug metabolism and excretion in urinary metabolites. Saliva microbiomics contributed the least.

**FIGURE 8:**
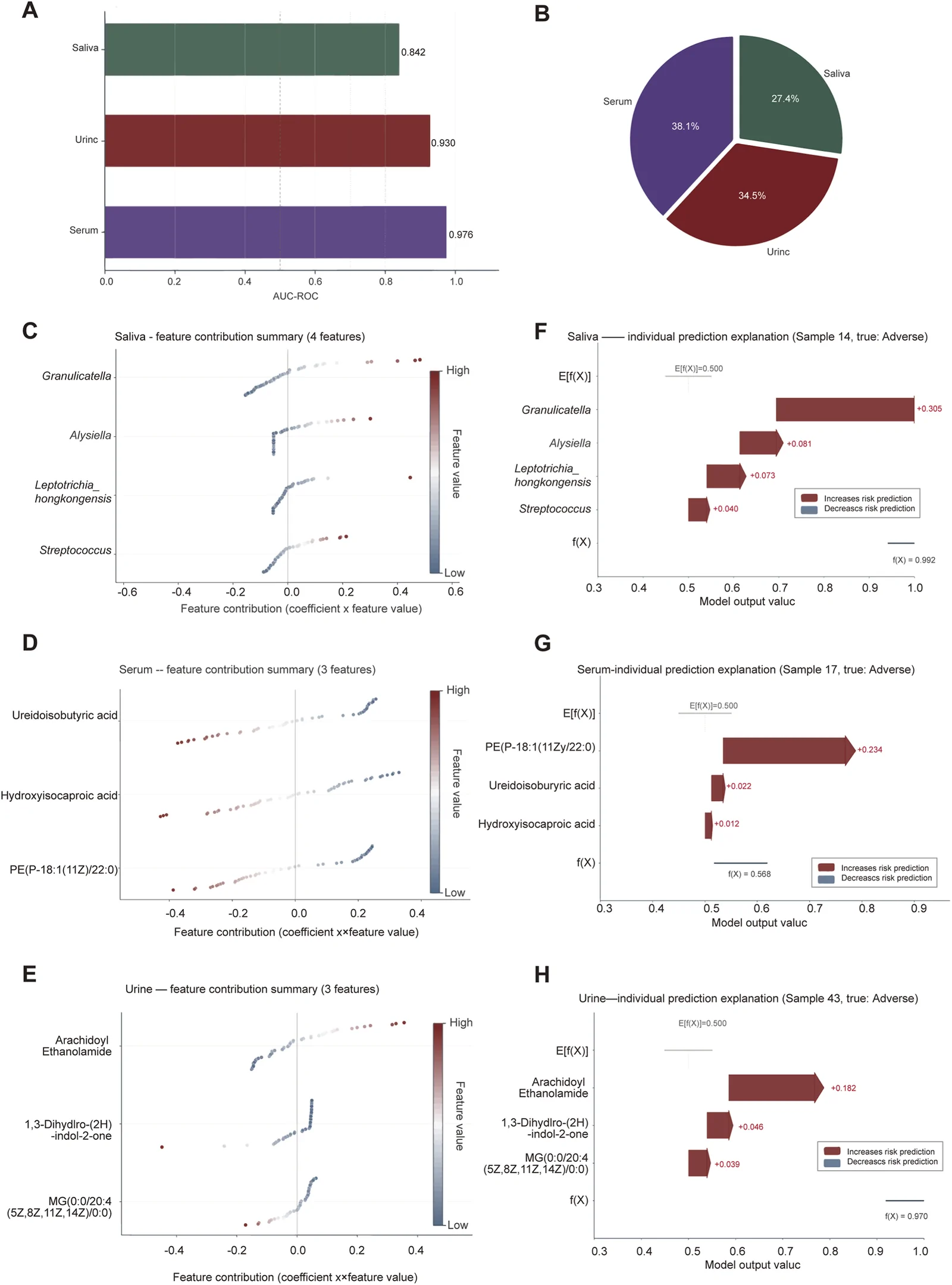
Screening of key metabolites and construction of predictive models **(A)** AUC-ROC comparison of models deployed across various omics; **(B)** pie chart of relative contribution based on AUC; **(C)** summary plot of salivary microbiome feature contributions; **(D)** summary plot of serum metabolome feature contributions (Beeswarm Plot); **(E)** summary plot of urine metabolome feature contributions; **(F)** individualized prediction waterfall plot for typical high-risk samples of salivary microbiota; **(G)** individualized prediction waterfall plot for typical high-risk samples of serum; **(H)** individualized prediction waterfall plot for typical high-risk samples of urine.

Furthermore, leveraging models deployed in various omics research projects, we extracted feature importance and conducted a contribution analysis. In the salivary microbiome random forest model, four model-associated discriminative features were identified: *Granulicatella*, *Alysiella*, *Leptotrichia hongkongensis*, and *Streptococcus*. Higher relative abundance of these genera increased the model’s predicted probability of adverse reactions (Figure 8C). In the serum metabolomics logistic regression deployment model, three key metabolites were identified: Ureidoisobutyric acid, Hydroxyisocaproic acid, and PE (P-18:1 (11Z)/22:0). All regression coefficients of these metabolites were negative, indicating higher metabolite concentrations yielded lower predicted adverse reaction probability within the classifier (Figure 8D). The three model-associated discriminative features extracted from the urinary LightGBM model included 1,3-Dihydro-(2H)-indol-2-one (2-indolinone, a downstream metabolite of the tryptophan-kynurenine pathway), Arachidoyl Ethanolamide and MG (0:0/20:4 (5Z,8Z,11Z,14Z)/0:0) (2-arachidonoylglycerol). Among these, Arachidoyl Ethanolamide displayed a positive weight in model prediction, meaning higher levels corresponded to higher predicted adverse reaction probability (Figure 8E). The other two metabolites represented features negatively correlated with predicted risk.

Based on the multi-omics, multi-model analysis described above, this study constructed a weighted evaluation formula to quantify the risk of adverse reactions for individual patients. Expanding all 10 specific characteristic variables, the complete evaluation formula for individual patient’s adverse reaction risk is as follows:

$$P_{\text{ADR}}=0.381\cdot f_{\text{LR}}\left(\frac{x_{S,1}-\mu_{S,1}}{\sigma_{S,1}},\frac{x_{S,2}-\mu_{S,2}}{\sigma_{S,2}},\frac{x_{S,3}-\mu_{S,3}}{\sigma_{S,3}}\right)+0.345\cdot \sigma \left(\beta_{0}+\beta_{1}\frac{x_{U,1}-\mu_{U,1}}{\sigma_{U,1}}+\beta_{2}\frac{x_{U,2}-\mu_{U,2}}{\sigma_{U,2}}+\beta_{3}\frac{x_{U,3}-\mu_{U,3}}{\sigma_{U,3}}\right)+0.274\cdot f_{\text{RF}}\left(\frac{x_{M,1}-\mu_{M,1}}{\sigma_{M,1}},\frac{x_{M,2}-\mu_{M,2}}{\sigma_{M,2}},\frac{x_{M,3}-\mu_{M,3}}{\sigma_{M,3}},\frac{x_{M,4}-\mu_{M,4}}{\sigma_{M,4}}\right)$$

The specific meanings of each variable are as follows.

**Table:** 

Symbol	Meaning
$x_{S,1},x_{S,2},x_{S,3}$ x_S,1 … x_S,3	Raw serum metabolite measurements (PE (P-18:1/22:0), α-hydroxyisocaproic acid, ureidoisobutyric acid)
$x_{U,1},x_{U,2},x_{U,3}$ x_U,1 … x_U,3	Raw urine metabolite measurements (2-indolinone, arachidoyl ethanolamide, 2-arachidonoylglycerol)
$x_{M,1},x_{M,2},x_{M,3},x_{M,4}$ x_M,1 … x_M,4	Relative abundances of salivary microorganisms (*Streptococcus*, *L. hongkongensis*, *Granulicatella*, *Alysiella*)
$\mu_{k,j},\sigma_{k,j}$ μ_k,j, σ_k,j	Mean and standard deviation of the corresponding feature in the training set
$f_{\text{LR}}\left(\cdot \right)$ f_S (·)	Output probability of the deployed serum logistic regression model (AUC = 0.976)
$\sigma \left(\cdot \right)$ σ(x)	Sigmoid function $\sigma \left(t\right)=1/\left(1+e^{-t}\right)$ , used only for linear models
$\beta_{0},\beta_{1},\beta_{2},\beta_{3}$ β_0^U, β_j^U	Feature importance weights (normalized) of the deployed urine LightGBM model
$f_{\text{RF}}\left(\cdot \right)$ f_M(·)	Output probability of the deployed salivary random forest model (AUC = 0.842)
0.381,0.345,0.274 w_S, w_U, w_M	AUC-based omics normalization weights: 38.1%/ 34.5%/ 27.4%

$$z_{k,j}=\frac{x_{k,j}-\mu_{k,j}}{\sigma_{k,j}},j=1,2,\ldots ,n_{k}$$

$$\omega_{k}=\frac{{\text{AUC}}_{k}-0.5}{\sum_{k^{\prime }=1}^{3}\left({\text{AUC}}_{k^{\prime }}-0.5\right)}$$

$$P_{\text{ADR}}=0.381\cdot P_{S}+0.345\cdot P_{U}+0.274\cdot P_{M}$$

$$P_{\text{ADR}}=0.381\cdot f_{\text{LR}}\left(\frac{x_{S,1}-\mu_{S,1}}{\sigma_{S,1}},\frac{x_{S,2}-\mu_{S,2}}{\sigma_{S,2}},\frac{x_{S,3}-\mu_{S,3}}{\sigma_{S,3}}\right)+0.345\cdot \sigma \left(\beta_{0}+\beta_{1}\frac{x_{U,1}-\mu_{U,1}}{\sigma_{U,1}}+\beta_{2}\frac{x_{U,2}-\mu_{U,2}}{\sigma_{U,2}}+\beta_{3}\frac{x_{U,3}-\mu_{U,3}}{\sigma_{U,3}}\right)+0.274\cdot f_{\text{RF}}\left(\frac{x_{M,1}-\mu_{M,1}}{\sigma_{M,1}},\frac{x_{M,2}-\mu_{M,2}}{\sigma_{M,2}},\frac{x_{M,3}-\mu_{M,3}}{\sigma_{M,3}},\frac{x_{M,4}-\mu_{M,4}}{\sigma_{M,4}}\right)$$

$$\text{Risk}=\left\{\begin{array}{ll} \text{High Risk}, & P_{\text{ADR}}\geq 0.597\\ \text{Low Risk}, & P_{\text{ADR}}<0.597 \end{array}\right.$$

To further elucidate the model’s predictive rationale for individual samples, this study conducted a waterfall chart analysis using representative high-risk adverse reaction samples. In the random forest model of the saliva microbiome, the prediction probability for samples in the adverse reaction group reached 0.992, with *Granulicatella* contributing the most (+0.305), significantly enhancing the prediction accuracy (Figure 8F). In the serum model, the predicted probability for this sample was 0.568. PE (P-18:1 (11Z)/22:0), ureidoisobutyric acid, and hydroxyisocaproic acid collectively contributed to the model’s risk output. However, its predicted probability approached the threshold, placing this sample in a borderline high-risk category (Figure 8G). Similarly, among urinary metabolites, the prediction probability was 0.970, with Arachidoyl Ethanolamide contributing to the sample being classified as high-risk (Figure 8H). This individualized analytical framework may help clinicians gain an intuitive understanding of each patient’s predictive profile and provide preliminary information to support individualized medication decisions.

## 4 Discussion

In clinical practice, the adverse reactions of mildly toxic traditional Chinese medicines are often overlooked. Because such herbs can exert important therapeutic effects in multiple diseases, predicting their adverse reactions is crucial. This study takes the commonly used mildly toxic medicinal herb EVR in the gastrointestinal tract as an example and finds that some patients experience adverse reactions after taking EVR-containing compound formulas. We collected samples from eligible patients, integrated serum metabolomics, urine metabolomics, and saliva microbiome data, and machine learning algorithms were applied to build a classification model for adverse event prediction. This study identified systemic metabolic shifts that co-occurred in patients who reported adverse reactions after receiving EVR-containing compound formulas, providing preliminary exploratory clues.

Through untargeted metabolomics analysis of serum and urine, it was suggested that in these two body fluids, the adverse reaction group mainly exhibited disturbances in amino acid metabolism. Significant changes were observed in arginine/proline metabolism, branched-chain amino acid biosynthesis, and tryptophan metabolism pathways. Moreover, most amino acid intermediates showed an upregulation trend, while some branched-chain amino acid decomposition products and downstream metabolites of tryptophan were downregulated in the adverse reaction group. Amino acids are the fundamental substances for energy metabolism, substance synthesis, and signal transduction. The maintenance of amino acid metabolism homeostasis is crucial for cellular function and tissue integrity. In neuroactive ligand-receptor interaction and dopaminergic synaptic pathways, levels of excitatory neurotransmitters such as glutamate and acetylcholine increased, whereas levels of inhibitory neurotransmitters (e.g., dopamine) and hormones (e.g., cortisol) decreased. These neural pathways have been reported to potentially modulate gastrointestinal sensation (Mittal et al., 2017; Zheng and Tao, 2025). These divergent neurotransmitter levels might coincide with upper gastrointestinal discomfort symptoms. At the same time, ABC transporter and mineral absorption pathways showed elevated overall signal intensity in affected patients, consistent with altered transmembrane transport of amino acids, carbohydrates, and minerals. Similar to metabolomics, the relative abundance of *Granulicatella*, *Alysiella*, *Leptotrichia hongkongensis*, and *Streptococcus* significantly increased in the oral microbiota of the adverse reaction group. Salivary microbiota is associated with various upper gastrointestinal diseases, including symptoms of gastroesophageal reflux and esophagitis, among others (Power et al., 2024; Sadeghi et al., 2025). Furthermore, research has demonstrated that oral microbiota might correlate with systemic inflammation and resultant whole-body metabolic dysfunction via direct translocation as well as the modulation of relevant metabolic signaling and immune metabolism (Gan et al., 2026).

Based on the omics results, we performed a side-by-side comparison of seven machine learning algorithms and selected the optimal prediction model for each dataset. Based on these optimal models, we then selected the characteristic metabolites or microbial communities for each omics modality. Three model-associated discriminative features were screened from serum data: PE (P-18:1 (11Z)/22:0), Hydroxyisocaproic acid, Ureidoisobutyric acid. PE is a phosphatidylethanolamine and a key phospholipid found in cell membranes and lipoproteins (Santos et al., 2024). The amphipathic nature of PE helps maintain cell membrane stability. PE acts as a lipid chaperone facilitating the folding of specific membrane proteins and supports the function of multiple respiratory complexes. PE concentration differences might correlate with altered cellular homeostasis-related metabolic signatures and are implicated in diverse pathological processes (Patel and Witt, 2017). Hydroxyisocaproic acid is a metabolic intermediate of leucine. The degradation of branched-chain amino acids mainly occurs in the mitochondrial matrix, participating in energy metabolism, mitochondrial biosynthesis, and protein quality control in mitochondria and cytoplasm (Li and Hoppe, 2023). Elevated levels of branched-chain amino acids might lead to mitochondrial dysfunction, thereby regulating energy metabolism and inflammation mechanisms in the body (Ye et al., 2020). Reduced Hydroxyisocaproic acid levels correlated with impaired mitochondrial branched-chain amino acid signatures, accompanied by abnormal cellular energy status. Ureidoisobutyric acid is derived from the pyrimidine degradation pathway, its reduced abundance correlates with disrupted nucleotide metabolism signatures that could tentatively link to DNA replication stress (Basbous et al., 2020). Variations of these three serum metabolites might correspond to concurrent shifts in membrane stability, energy turnover and cellular maintenance processes. The three urinary substances could reflect changes related to neuroactive molecules. 2-Indolinone is a downstream product of the tryptophan-kynurenine pathway. The metabolism of tryptophan via the kynurenine pathway is critical for immune function. Cell-protective and immunomodulatory properties of the kynurenine pathway in conditions affecting the central or peripheral nervous system and other diseases such as inflammatory bowel disease (Sheibani et al., 2023). Fluctuations in the abundance of this metabolite may accompany a relative rise of neurotoxic metabolites, alongside changes related to gastrointestinal neural activity. Arachidoyl Ethanolamide and 2-arachidonoylglycerol are endogenous cannabinoid-like molecules. The endocannabinoid system consists of receptors, endocannabinoids, and enzymes that regulate biosynthesis and degradation, playing a fundamental role in the physiology and pathology of the gastrointestinal tract (Aloisio Caruso et al., 2025). Elevated Arachidoyl Ethanolamide correlated with signatures consistent with heightened endocannabinoid signaling, which may associate with altered gastrointestinal motility and visceral sensation. 2-Arachidonoylglycerol has been associated with many pathological conditions, including obesity, metabolic syndrome, liver disease, pain, neurological disorders, and inflammation (Turcotte et al., 2015). Variations in 2-arachidonoylglycerol concentrations may emerge together with metabolic profiles hinting at inflammatory signals associated with upper gastrointestinal discomfort. Four salivary genera, *Granulicatella*, *Alysiella*, *Leptotrichia hongkongensis*, *Streptococcus*, that served as model-associated discriminative features were significantly enriched in patients with adverse reactions. They are all opportunistic pathogens. *Granulicatella* belongs to the normal oral, gastrointestinal, and urogenital microbiota, but they may cause serious infections in individuals with normal or low immune function. *Granulicatella* genus can also cause lung infections, gastrointestinal sepsis, etc .,(Bokhari et al., 2025). *Alysiella* is a member of the *Neisseriaceae* family, which mainly colonizes the oral and pharyngeal regions of animals and humans. Overabundance of these genera might correlate with local inflammatory cascade signatures under dysbiosis status. *Leptotrichia hongkongensis*, as a normal commensal bacterium in the oral and intestinal tract, is an opportunistic pathogen that can enter the circulatory system through translocation and induce bacteremia (Eribe and Olsen, 2017). *Streptococcus* may lead to excessive secretion of inflammatory cytokines, disruption of the host immune system (Qi et al., 2022). Abundance shifts of these four microbial taxa may align with the altered amino acid and neuroactive metabolite profiles detected in this cohort, and such parallel changes could potentially relate to the emergence of upper gastrointestinal adverse symptoms. Even so, these findings merely represent preliminary observational clues, and dedicated follow-up verification is still required to validate the above speculations.

We also employed an AUC-weighted fusion method to derive a combined probability prediction formula, with a combined threshold of 0.597. This formula integrates the classification contribution of 10 model-associated discriminative features to calculate individual predicted adverse reaction probability, providing an exploratory multi-omics quantitative prediction framework for statistical risk stratification. The model cannot be directly applied to routine clinical practice at present, and large-sample verification is required before clinical translation. Among various omics, serum metabolomics ranks first in terms of contribution. Saliva collection is fast and completely non-invasive, giving it a clear advantage for rapid outpatient screening. The urine model has excellent performance, with a specificity of 1.000 and a balanced accuracy of 0.943, making it suitable for safe medication monitoring scenarios.

The study has certain limitations. Multi-omics analyses were performed on partially overlapping subsets from the total 86 enrolled patients, with 52 samples for serum metabolomics, 48 for urine metabolomics and 56 for salivary microbiome. Such relatively small and uneven sample subsets render machine-learning-based prediction models vulnerable to overfitting. Although we adopted LOOCV with nested feature-selection workflow to minimize information leakage and maximize usage of limited samples, it should be acknowledged that performance estimates obtained from small-sample datasets may remain optimistic even with LOOCV. These results should therefore be regarded as preliminary exploratory findings until validated in independent cohorts. In addition, all participants received EVR-containing compound formulas rather than isolated EVR. Consequently, co-administered herbal ingredients, underlying disease status, and concomitant medications represent confounding factors that cannot be fully excluded. Although EVR is the only ingredient documented as mildly toxic in classical Chinese medicine records and modern toxicological studies, and experienced clinicians considered it a plausible contributor to the observed adverse events, this hypothesis cannot be conclusively validated by the current observational design and remains an unproven assumption. This cohort can only identify statistical associations between omics signatures and adverse reactions, all pathway-related inferences are therefore tentative hypotheses requiring experimental verification, and independent external validation in larger cohorts is necessary. In addition, microbial and metabolic signals may be influenced by external factors such as diet and oral hygiene, and residual confounding cannot be entirely excluded despite our efforts to control for these factors. Future studies should record exposure details more comprehensively and integrate pharmacokinetic data or individual exposure levels, as well as exposure biomarkers and dynamic follow-up data, to enhance the interpretability and translational potential of the findings.

## 5 Conclusion

This observational study identified systemic molecular and microbial signatures that may correlate with adverse events in patients exposed to EVR-containing compound formulas, including shifts in amino acid metabolism, neuroactive molecule homeostasis, bile secretion pathways, and oral microbial composition. Among the serum metabolites, three model-associated discriminative features were identified: PE (P-18:1/22:0), Hydroxyisocaproic acid, and Ureidoisobutyric acid. Among the urinary metabolites, 1, 3-Dihydro-(2H)-indol-2-one, MG (0:0/20:4/0:0), and Arachidoyl Ethanolamide were identified. In the salivary microbiome, *Granulicatella*, *Alysiella*, *Leptotrichia hongkongensis*, and *Streptococcus* were identified as key microbial communities. A multi-omics integrated prediction formula was established using an AUC-weighted fusion strategy. A score ≥ 0.597 indicates a higher model-predicted probability of adverse gastrointestinal outcomes among patients receiving EVR-containing compound formulas. The above model enables statistical quantification of predicted adverse reaction probability for individual patients and provides preliminary reference for safety evaluation and risk stratification of low-toxicity herbal medicines.

## Data Availability

The original contributions presented in the study are included in the article/Supplementary Material. The raw 16S rRNA amplicon sequencing data are not publicly available due to restrictions imposed by Chinese laws and regulations on human genetic resources and by the terms of the informed consent obtained from the participants. De-identified processed data, including the 16S rRNA ASV/OTU abundance table and de-identified metabolomics data, are available from the corresponding author upon reasonable request, subject to applicable legal and ethical requirements.

## Glossary

16S rRNA: 16S Ribosomal RNA
2-AG: 2-Arachidonoylglycerol
ABC: ATP-Binding Cassette
ACE: Abundance-Based Coverage Estimator
ANOSIM: Analysis of Similarities
ANOVA: Analysis of Variance
ASV: Amplicon Sequence Variant
AUC: Area Under the Curve
AUC-PR: Area Under the Precision–Recall Curve
AUC-ROC: Area Under the Receiver Operating Characteristic Curve
CI: Confidence Interval
COG: Clusters of Orthologous Groups
EC: Enzyme Commission
ECS: Endocannabinoid System
EVR: Evodia rutaecarpa/ Evodiae Fructus
FDR: False Discovery Rate
HPLC: High-Performance Liquid Chromatography
HRMS: High-Resolution Mass Spectrometry
IBD: Inflammatory Bowel Disease
KEGG: Kyoto Encyclopedia of Genes and Genomes
KNN: k-Nearest Neighbor
LASSO: Least Absolute Shrinkage And Selection Operator
LC-MS: Liquid Chromatography–Mass Spectrometry
LC-MS/MS: Liquid Chromatography–Tandem Mass Spectrometry
LDA: Linear Discriminant Analysis
LEfSe: Linear Discriminant Analysis Effect Size
LOOCV: Leave-One-Out Cross-Validation
LR: Logistic Regression
MS/MS: Tandem Mass Spectrometry
NMDS: Non-Metric Multidimensional Scaling
NPV: Negative Predictive Value
OPLS-DA: Orthogonal Partial Least Squares Discriminant Analysis
OTU: Operational Taxonomic Unit
PCA: Principal Component Analysis
PCoA: Principal Coordinate Analysis
PCR: polymerase chain reaction
PE: Phosphatidylethanolamine
PICRUSt2: Phylogenetic Investigation of Communities by Reconstruction of Unobserved States 2
PLS-DA: Partial Least Squares Discriminant Analysis
PPV: Positive Predictive Value
PQN: Probabilistic Quotient Normalization
QC: Quality Control
ROC: Receiver Operating Characteristic
RSD: Relative Standard Deviation
SD: Standard Deviation
SHAP: SHapley Additive ExPlanations
SVM: Support Vector Machine
TIC: Total Ion Chromatogram
UHPLC: Ultra-High-Performance Liquid Chromatography
UHPLC-HRMS: Ultra-High-Performance Liquid Chromatography–High-Resolution Mass Spectrometry
VIP: Variable Importance in Projection.
