# Supplementary material for: Multi-omics and machine learning based on clinical samples: revealing the toxicity characteristics in adverse reactions to Evodia rutaecarpa
Source: Front Pharmacol. 2026 Oct 19;17:1901889. doi: 10.3389/fphar.2026.1901889 (PMC13724504; doi:10.3389/fphar.2026.1901889)
Supplement: Supplementary file 1 [file Supplementaryfile1.docx]

**Supplementary material: analysis of *Evodia rutaecarpa* aqueous extract and machine learning methodology**

***Methods***

**1 Preparation and Chromatographic Analysis of Water Extract of EVR**

**1.1 Preparation of water extract of EVR**

The dried fruit of *Evodia rutaecarpa* (Juss.) Benth meets the standards of the Chinese Pharmacopoeia. Preparation process: Weigh the raw medicinal material of EVR, grind it into coarse powder, soak it in 6 times the amount of water for 30 minutes, heat and boil it twice, each time for 1 hour, filter the residue through a 200 mesh sieve, combine the filtrate twice, concentrate the filtrate with a rotary evaporator, dry, grind, and store it in a -20 ℃ refrigerator.

**1.2 EVR high-performance liquid chromatography**

Dissolve EVR powder in 50 mL of methanol and extract with ultrasound for 30 min (250 W, 40 KHz). Filter through a 0.22 μm microporous membrane and take the subsequent filtrate as the test solution. The solution was filtered through a 0.22 μm membrane and then analyzed on a Shim‑pack GIST C18 column (250 mm × 4.6 mm, 5 μm). The mobile phase is ultrapure water containing 0.085% phosphoric acid (A) and acetonitrile (B), and the gradient elution procedure is as follows: 0-25 min, 4-14% B; 25-26 minutes, 14-16% B; 26-46 minutes, 16-20% B; 46-56 minutes, 20-40% B; 56-70 minutes, 40-50% B. The column temperature is 35 ° C, the flow rate is 0.6 mL/min, and the injection volume is 10 μL. The spectrum is recorded at 270 nm for 0-56 minutes and the wavelength is changed to 240 nm within 56-70 minutes.

**2 Machine learning method**

**2.1 Removal of exogenous metabolites**

To eliminate the interference of drug metabolites, synthetic chemicals, and environmental/dietary pollutants in predicting adverse reactions, this study first excluded exogenous metabolites from serum and urine metabolomics before entering univariate statistical screening.

**2.2 Feature pre screening**

Use Welch's t-test for univariate feature screening. For each feature, calculate the t-statistic and corresponding p-value between the two groups. Feature filtering is independently performed on the training set of each fold of LOOCV. Directly select the top 20-40 features sorted by p-value as candidates for subsequent L1 embedding selection, in order to maximize the preservation of real biological signals.

**2.3 Regularized logistic regression model**

Construct a prediction model using a two-stage regularization strategy. The first stage uses L1 regularization (LASSO) for embedded feature selection, which automatically compresses the coefficients of unimportant features to zero by adding a penalty term for the absolute value of the coefficients in the loss function. In the second stage, based on the features selected by L1, L2 regularization (Ridge) is used to construct the final prediction model to improve model stability. The regularization intensity parameter is recalibrated in v2 (after exogenous exclusion): serum metabolomics uses C_select=0.002 and C_final=0.015; Urine metabolomics uses C_select=0.010 and C_final=0.050; The saliva microbiome uses C_select=0.3 and C_final=0.8.

**2.4 Horizontal comparison of multiple models**

To evaluate the predictive performance of different machine learning algorithms on small sample multi‑omics data, this study conducted a horizontal comparison of seven commonly used classification models based on a unified feature selection process (Welch's t-test pre screening → L1 regularized embedded selection). Each model shares the same selected features (3 in serum and urine, 4 in saliva) at each fold in LOOCV, only replacing the final classifier to ensure fairness in comparison. The 7 candidate models and their hyperparameter configurations optimized for small sample scenarios are as follows:

**Candidate Models and Hyperparameter Configuration**

| **model** | **Key hyperparameters** | **Category imbalance handling** |
| --- | --- | --- |
| Logistic Regression (L2 Regularization) | C=0.012-0.8 (adjusted by omics) | class_weight='balanced' |
| Support Vector Machine (RBF Kernel) | C=1.0, gamma='scale' | class_weight='balanced' |
| Random Forest | n_estimators=100, max_depth=3, min_samples_leaf=3 | class_weight='balanced' |
| XGBoost | n_estimators=50, max_depth=2, reg_alpha=1.0, reg_lambda=2.0 | Dynamic calculation of scale_pos_weight |
| LightGBM | n_estimators=50, max_depth=2, num_leaves=4, min_child_samples=3 | is_unbalance |
| K-Nearest Neighbor (KNN) | n_neighbors=5, weights='distance' | Distance weighted |
| Naive Bayes (Gaussian) | priors=[0.5, 0.5] | Prior equalization |

The selection of hyperparameters follows the following principles: (1) Tree models (random forest, XGBoost, LightGBM) adopt shallow structures (max_depth=2-3) to prevent overfitting of small samples; (2) All models that support category weights enable a balanced strategy; (3) The regularization strength tends to be conservative, prioritizing the guarantee of generalization ability. Each model selects the optimal classifier for each omics based on AUC-ROC, and comprehensively considers the stability of probability output to determine the final deployment model. Subsequent performance evaluation, feature importance analysis, and personalized prediction interpretation are all based on the deployment models of each omics.

**2.5 Leave-one-out cross validation**

Considering the limited sample size, this study adopts the LOOCV method to evaluate the performance of the model. The basic process of LOOCV is to use one sample as the test set and the remaining samples as the training set, independently completing all steps of feature screening, standardization, L1 feature selection, and classifier training on the training set, and then predicting the test samples. Repeat n times (n is the total number of samples), and finally summarize the predicted results of all samples to calculate performance indicators. All feature selection steps are performed within the cross validation loop to avoid information leakage. It should be noted that LOOCV cannot fully eliminate optimistic bias for small‑sample data. Therefore, these findings remain preliminary pending independent‑cohort validation.

**2.6 Category imbalance handling**

Due to the imbalanced sample size between the adverse reaction group and the control group, this study adopted a category weight adjustment strategy (class_weight='balanced') in model training to give higher weights to minority classes and balance the classifier's attention to both classes

**2.7 Optimal threshold determination and performance evaluation**

The Youden's J statistic (J=Sensitivity+Specificity -1) is used to determine the optimal classification threshold, which seeks the best balance between sensitivity and specificity. The performance evaluation indicators of the model include: AUC-ROC (area under the receiver operating characteristic curve), sensitivity, specificity, positive predictive value (PPV), negative predictive value (NPV), balance accuracy, and Brier score.

**2.8 Feature importance and omics weight analysis**

To explain the prediction mechanism of the model, this study adopts corresponding feature importance extraction methods for different types of deployment models. For linear models (logistic regression), regression coefficients are directly used as feature weights, and the sign and magnitude of the coefficients reflect the direction and contribution strength of the features, respectively. For tree models (LightGBM, random forest), the built-in split based/Gini importance is used, and the contribution direction is determined by the Pearson correlation between feature values and predicted probabilities (positive correlation is the risk factor, negative correlation is the protection factor).

The omics weights are calculated based on the AUC values of the omics deployment models. The specific method is to calculate the part of each omics AUC that exceeds the random classification (0.5), and then normalize it to obtain the relative contribution. This method reflects the relative importance of omics data in predicting adverse reactions.

**2.9 Individualized prediction interpretation**

To achieve personalized prediction interpretation, this study calculated the feature contribution values of each sample. For linear models (logistic regression), feature contribution is defined as the product of the standardized feature values of the sample and the corresponding regression coefficients, with precise additive decomposition properties. For nonlinear models (LightGBM, Random Forest), feature contribution is defined as the product of standardized feature values and normalized feature importance weights, as an approximate decomposition. This study uses visualization methods similar to SHAP (SHapley Additive exPlanations) (beeswarm plot and waterfall plot) to display feature contributions, in order to visually present the risk factor composition of individual patients. It should be noted that for nonlinear models, this approximate decomposition does not possess the exact additive property of SHAP values, but can still provide meaningful feature contribution directions and relative size references.

***Results***

**1. Risk-weighted Assessment Formula for Adverse Reactions**

Based on the results of multi-omics and multi-model analysis, this study established a comprehensive weighted risk assessment framework for adverse drug reactions (ADRs), enabling quantitative evaluation of individual patient ADR risks.

**1.1 Feature Standardization**

For the k-th omics dataset ($\text{k}\text{∈\{}\text{S}\text{,}\text{U}\text{,}\text{M}\text{\}}$，Representing Serum, Urine, and saliva Microbiome respectively), given the patient's original feature measurement values $\text{x}_{\text{k}\text{,}\text{j}}$, First, perform Z-score normalization.:

$$\text{z}_{\text{k}\text{,}\text{j}}\text{=}\frac{\text{x}_{\text{k}\text{,}\text{j}}\text{-}\text{μ}_{\text{k}\text{,}\text{j}}}{\text{σ}_{\text{k}\text{,}\text{j}}}\text{, }\text{j}\text{=1,2,…,}\text{n}_{\text{k}}$$

$\text{μ}_{\text{k}\text{,}\text{j}}$ and $\text{σ}_{\text{k}\text{,}\text{j}}$ represent the mean and standard deviation of the j-th feature in the k-th omics dimension within the training set, respectively.

**1.2 Single-Omics Predictive Model**

Each omics deployment model outputs the probability of adverse reaction risk based on standardized feature vectors:

$$\text{P}_{\text{k}}\text{=}\text{f}_{\text{k}}\text{(}\text{z}_{\text{k}}\text{), }\text{k}\text{∈\{}\text{S}\text{,}\text{U}\text{,}\text{M}\text{\}}$$

Here, f_S represents the Logistic Regression model (serum data with 3 endogenous features), f_U denotes the LightGBM model (urine data with 3 endogenous features), and f_M stands for the Random Forest model (saliva data with 4 microbial taxa).

For the serum Logistic Regression deployment model, the predicted probability follows an explicit analytical sigmoid form; whereas the urine LightGBM and saliva Random Forest models are tree ensemble models, with predicted probabilities obtained through leaf node averaging or probability calibration.

$$\text{P}_{\text{U}}\text{=}\text{σ}\text{​}\left( \text{β}_{\text{0}}\text{+}\sum_{\text{j}\text{=1}}^{\text{3}} \text{β}_{\text{j}}\text{⋅}\text{z}_{\text{U}\text{,}\text{j}} \right)\text{=}\frac{\text{1}}{\text{1+exp​[-(}\text{β}_{\text{0}}\text{+}\text{β}_{\text{1}}\text{z}_{\text{U}\text{,1}}\text{+}\text{β}_{\text{2}}\text{z}_{\text{U}\text{,2}}\text{+}\text{β}_{\text{3}}\text{z}_{\text{U}\text{,3}}\text{)]}}$$

**1.3 Feature Contribution Decomposition**

For linear models, the contribution of the j-th feature can be precisely decomposed, for nonlinear models, an approximate decomposition of normalized feature importance is employed:

$$\text{C}_{\text{k}\text{,}\text{j}}\text{=}\text{w}_{\text{k}\text{,}\text{j}}\text{⋅}\text{z}_{\text{k}\text{,}\text{j}}$$

In the linear model (serum logistic regression), β_j represents the L2-regularized regression coefficient; in the nonlinear models (urine LightGBM and saliva random forest), β_j denotes the normalized split-based/Gini importance. $\text{w}_{\text{k}\text{,}\text{j}}\text{=}\text{β}_{\text{k}\text{,}\text{j}}\text{w}_{\text{k}\text{,}\text{j}}$

The specific characteristics and weights of each omics group are shown in the table below:

| Omics | Variable symbol | Feature Name | The weight of $\text{w}_{\text{k}\text{,}\text{j}}$ | Direction |
| --- | --- | --- | --- | --- |
| serum (S) | $\text{x}_{\text{S}\text{,1}}$x_S,1 | PE(P-18:1(11Z)/22:0) | -0.197 | Protect |
| serum (S) | $\text{x}_{\text{S}\text{,2}}$x_S,2 | Hydroxyisocaproic acid | -0.203 | Protect |
| serum (S) | $\text{x}_{\text{S}\text{,3}}$x_S,3 | Ureidoisobutyric acid | -0.208 | Protect |
| Urine (U) | $\text{x}_{\text{U}\text{,1}}$x_U,1 | 1,3-Dihydro-(2H)-indol-2-one | -0.089 | Protect |
| Urine (U) | $\text{x}_{\text{U}\text{,2}}$x_U,2 | Arachidoyl Ethanolamide | +0.138 | Risk |
| Urine (U) | $\text{x}_{\text{U}\text{,3}}$x_U,3 | MG(0:0/20:4(5Z,8Z,11Z,14Z)/0:0) | -0.055 | Protect |
| Saliva (M) | $\text{x}_{\text{M}\text{,1}}$x_M,1 | Streptococcus | +0.074 | Risk |
| Saliva (M) | $\text{x}_{\text{M}\text{,2}}$x_M,2 | Leptotrichia_hongkongensis | +0.074 | Risk |
| Saliva (M) | $\text{x}_{\text{M}\text{,3}}$x_M,3 | Granulicatella | +0.148 | Risk |
| Saliva (M) | $\text{x}_{\text{M}\text{,4}}$x_M,4 | Alysiella | +0.085 | Risk |

**1.4 Omics Weight Calculation**

The multi-omics integrated prediction employs an AUC-based weighted fusion strategy. The calculation method for each omics weight w_k involves normalizing (AUC_k−0.5); substituting the AUC values of the v2 omics deployment models yields: AUC_S = 0.976, AUC_U = 0.930, and AUC_M = 0.842, resulting in the final weights w_S=0.381（38.1%），w_U=0.345（34.5%），w_M=0.274（27.4%）。$\text{ω}_{\text{k}}$

$$\text{ω}_{\text{k}}\text{=}\frac{\text{AUC}_{\text{k}}\text{-0.5}}{\sum_{\text{k}\text{'=1}}^{\text{3}} \left( \text{AUC}_{\text{k}\text{'}}\text{-0.5} \right)}$$

AUC values for each omics deployment model:

$$\text{ω}_{\text{S}}\text{=}\frac{\text{0.976-0.5}}{\text{(0.976-0.5)+(0.930-0.5)+(0.842-0.5)}}\text{=}\frac{\text{0.476}}{\text{1.248}}\text{≈0.381}$$

$$\text{ω}_{\text{U}}\text{=}\frac{\text{0.930-0.5}}{\text{1.248}}\text{=}\frac{\text{0.430}}{\text{1.248}}\text{≈0.345}$$

$$\text{ω}_{\text{M}}\text{=}\frac{\text{0.842-0.5}}{\text{1.248}}\text{=}\frac{\text{0.342}}{\text{1.248}}\text{≈0.274}$$

**1.5 Multi-omics Combined Risk Probability**

The joint risk probability is the weighted sum of the independent prediction probabilities for each omics dataset:

$$\text{P}_{\text{ADR}}\text{=}\text{ω}_{\text{S}}\text{⋅}\text{P}_{\text{S}}\text{+}\text{ω}_{\text{U}}\text{⋅}\text{P}_{\text{U}}\text{+}\text{ω}_{\text{M}}\text{⋅}\text{P}_{\text{M}}\text{=0.381⋅}\text{P}_{\text{S}}\text{+0.345⋅}\text{P}_{\text{U}}\text{+0.274⋅}\text{P}_{\text{M}}$$

The combined classification threshold also employs weighted fusion:

$$\text{T}_{\text{ADR}}\text{=}\text{ω}_{\text{S}}\text{⋅}\text{T}_{\text{S}}\text{+}\text{ω}_{\text{U}}\text{⋅}\text{T}_{\text{U}}\text{+}\text{ω}_{\text{M}}\text{⋅}\text{T}_{\text{M}}\text{=0.381×0.495+0.345×0.627+0.274×0.700≈0.597}$$

Risk Assessment Rule:

$$\text{Risk Level=}\left\{ \begin{matrix} \text{High Risk,} & \text{if}\text{P}_{\text{ADR}}\text{≥0.597} \\ \text{Low Risk,} & \text{if}\text{P}_{\text{ADR}}\text{<0.597} \end{matrix} \right.$$

**1.6 Complete Evaluation Formula for Variables**

By integrating all the aforementioned steps and expanding the 10 specific characteristic variables, the complete assessment formula for individual patient adverse reaction risk is as follows:

$$\text{z}_{\text{k}\text{,}\text{j}}\text{=}\frac{\text{x}_{\text{k}\text{,}\text{j}}\text{-}\text{μ}_{\text{k}\text{,}\text{j}}}{\text{σ}_{\text{k}\text{,}\text{j}}}\text{, }\text{j}\text{=1,2,…,}\text{n}_{\text{k}}$$

$$\text{ω}_{\text{k}}\text{=}\frac{\text{AUC}_{\text{k}}\text{-0.5}}{\sum_{\text{k}\text{'=1}}^{\text{3}} \left( \text{AUC}_{\text{k}\text{'}}\text{-0.5} \right)}$$

$$\text{P}_{\text{ADR}}\text{=0.381⋅}\text{P}_{\text{S}}\text{+0.345⋅}\text{P}_{\text{U}}\text{+0.274⋅}\text{P}_{\text{M}}$$

$$\begin{matrix} \text{P}_{\text{ADR}}\text{= } & \text{0.381⋅}\text{f}_{\text{LR}}\text{​}\left( \frac{\text{x}_{\text{S}\text{,1}}\text{-}\text{μ}_{\text{S}\text{,1}}}{\text{σ}_{\text{S}\text{,1}}}\text{, }\frac{\text{x}_{\text{S}\text{,2}}\text{-}\text{μ}_{\text{S}\text{,2}}}{\text{σ}_{\text{S}\text{,2}}}\text{, }\frac{\text{x}_{\text{S}\text{,3}}\text{-}\text{μ}_{\text{S}\text{,3}}}{\text{σ}_{\text{S}\text{,3}}} \right) \\ \text{+ } & \text{0.345⋅}\text{σ}\text{​}\left( \text{β}_{\text{0}}\text{+}\text{β}_{\text{1}}\frac{\text{x}_{\text{U}\text{,1}}\text{-}\text{μ}_{\text{U}\text{,1}}}{\text{σ}_{\text{U}\text{,1}}}\text{+}\text{β}_{\text{2}}\frac{\text{x}_{\text{U}\text{,2}}\text{-}\text{μ}_{\text{U}\text{,2}}}{\text{σ}_{\text{U}\text{,2}}}\text{+}\text{β}_{\text{3}}\frac{\text{x}_{\text{U}\text{,3}}\text{-}\text{μ}_{\text{U}\text{,3}}}{\text{σ}_{\text{U}\text{,3}}} \right) \\ \text{+ } & \text{0.274⋅}\text{f}_{\text{RF}}\text{​}\left( \frac{\text{x}_{\text{M}\text{,1}}\text{-}\text{μ}_{\text{M}\text{,1}}}{\text{σ}_{\text{M}\text{,1}}}\text{, }\frac{\text{x}_{\text{M}\text{,2}}\text{-}\text{μ}_{\text{M}\text{,2}}}{\text{σ}_{\text{M}\text{,2}}}\text{, }\frac{\text{x}_{\text{M}\text{,3}}\text{-}\text{μ}_{\text{M}\text{,3}}}{\text{σ}_{\text{M}\text{,3}}}\text{, }\frac{\text{x}_{\text{M}\text{,4}}\text{-}\text{μ}_{\text{M}\text{,4}}}{\text{σ}_{\text{M}\text{,4}}} \right) \end{matrix}$$

$$\text{Risk=}\left\{ \begin{matrix} \text{High Risk,} & \text{P}_{\text{ADR}}\text{≥0.597} \\ \text{Low Risk,} & \text{P}_{\text{ADR}}\text{<0.597} \end{matrix} \right.$$

**2. Component analysis results of EVR**

**Table S1. Metabolites identified in EVR via LC‒MS analysis**

| Name | tR (min) | Quasimoleculary (Error,ppm)[M-H]- | Quasimoleculary (Error,ppm)  [M+H]+ | δ/ppm | Molecular formula | m/z Calculatede | MS/MS Fragments |
| --- | --- | --- | --- | --- | --- | --- | --- |
| trans-Cinnamaldehyde | 9.047 |  | 133.06499 | 1.12 | C9H8O | 132.05766 | 131.1 105.0 103.0 |
| rutaecarpine | 16.87 |  | 288.11316 | 0.07 | C18H13N3O | 287.10588 | 288.1 244.0 |
| Quercetin | 7.19 |  | 303.05008 | 0.50 | C15H10O7 | 302.0428 | 303.0 |
| Neochlorogenic acid | 3.61 | 353.08783 |  | -0.08 | C16H18O9 | 354.09505 | 191.0 179.0 |
| Isorhamnetin | 7.999 |  | 317.06537 | -0.67 | C16H12O7 | 316.05809 | 317.0 302.0 |
| Hesperidin | 8.317 | 611.19849 |  | 2.40 | C28H34O15 | 610.19124 | 177.0 |
| Gallic acid | 2.138 | 169.0136 |  | -3.83 | C7H6O5 | 170.02087 | 169.0 |
| dehydroevodiamine | 8.790 |  | 302.12936 | 1.81 | C19H15N3O | 301.12206 | 302.1 287.1 |
| Coumarin | 5.650 |  | 147.04427 | 0.08 | C9H6O2 | 146.03679 | 91.1 |
| Citric acid | 1.670 | 191.01929 |  | -2.20 | C6H8O7 | 192.02658 | 129.0 111.0 87.0 |
| Chlorogenic acid | 4.605 | 355.10205 |  | -0.50 | C16H18O9 | 354.09491 | 135.0 |
| Catechin | 4.728 | 289.07172 |  | -0.17 | C15H16O7 | 290.07899 | 245.0 123.0 |
| Caffeic acid | 4.466 |  | 181.04933 | -1.13 | C9H8O4 | 180.04206 | 135.0 |

**Fig. S1 UHPLC–HRMS Base Peak Chromatograms and Major Chemical Structures of the Aqueous Extract of EVR**

(A–B) UHPLC–HRMS base peak chromatograms of the aqueous extract of EVR in positive ion mode (A) and negative ion mode (B); (C–P) Molecular structure diagrams of the 13 major chemical constituents.

**Two primary types of EVR-containing herbal formulas:**

Formula 1

Evodia rutaecarpa 5 g, Coptidis Rhizoma 5 g, Ginseng Radix et Rhizoma 5 g, Magnoliae Officinalis Rhizoma Preparata cum Zingibere 10 g, Aurantii Fructus Immaturus 10 g, Atractylodis Macrocephalae Rhizoma 10 g, Poria Cocos 15 g, Perillae Folium 10 g, Hordei Fructus Germinatus Preparatus 15 g, Glycyrrhizae Radix et Rhizoma Preparata cum Melle 10 g.

Formula 2

Evodia rutaecarpa 6 g, Ginseng Radix et Rhizoma 9 g, Zingiberis Rhizoma Recens 18 g, Jujubae Fructus 10 g, Magnoliae Officinalis Rhizoma Preparata cum Zingibere 10 g, Hordei Fructus Germinatus Preparatus 30 g, Glycyrrhizae Radix et Rhizoma Preparata cum Melle 10 g.

These two formulas represent the core base prescriptions for this cohort. Most patients received the original formulas, whereas a subset of participants were administered modified variants with minor additive or subtractive herbal adjustments according to individual clinical conditions. All modified prescriptions retained *Evodia rutaecarpa* as a key component.
